# Dynamic Profiling and Binding Affinity Prediction of NBTI Antibacterials against DNA Gyrase Enzyme by Multidimensional Machine Learning and Molecular Dynamics Simulations

**DOI:** 10.1021/acsomega.4c00036

**Published:** 2024-04-11

**Authors:** Maja Kokot, Nikola Minovski

**Affiliations:** † Laboratory for Cheminformatics, Theory Department, National Institute of Chemistry, Hajdrihova 19, 1001 Ljubljana, Slovenia; ‡ The Department of Pharmaceutical Chemistry, Faculty of Pharmacy, University of Ljubljana, Aškerčeva cesta 7, 1000 Ljubljana, Slovenia

## Abstract

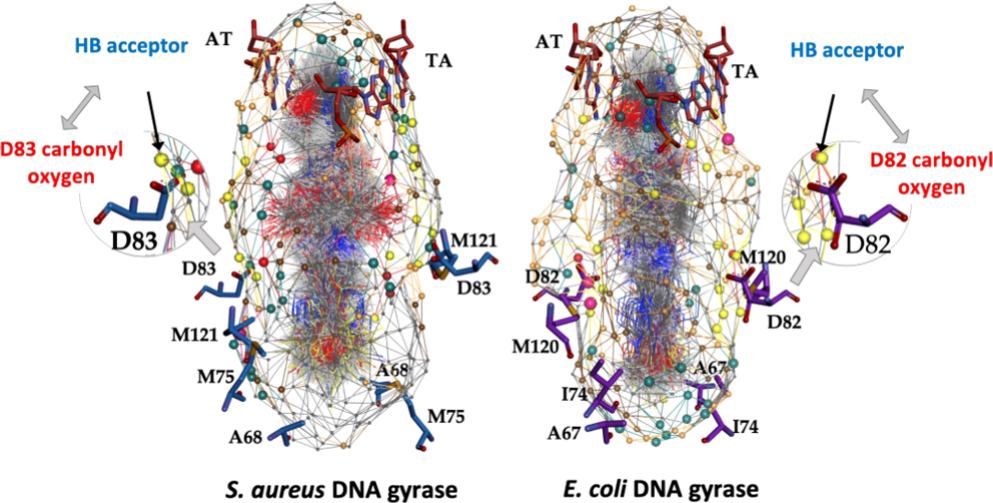

Bacterial type II topoisomerases are well-characterized and clinically important targets for antibacterial chemotherapy. Novel bacterial topoisomerase inhibitors (NBTIs) are a newly disclosed class of antibacterials. Prediction of their binding affinity to these enzymes would be beneficial for *de novo* design/optimization of new NBTIs. Utilizing *in vitro* NBTI experimental data, we constructed two comprehensive multidimensional DNA gyrase surrogate models for *Staphylococcus aureus* (*q*^2^ = 0.791) and *Escherichia coli* (*q*^2^ = 0.806). Both models accurately predicted the IC_50_s of 26 NBTIs from our recent studies. To investigate the NBTI’s dynamic profile and binding to both targets, 10 selected NBTIs underwent molecular dynamics (MD) simulations. The analysis of MD production trajectories confirmed key hydrogen-bonding and hydrophobic contacts that NBTIs establish in both enzymes. Moreover, the binding free energies of selected NBTIs were computed by the linear interaction energy (LIE) method employing an in-house derived set of fitting parameters (α = 0.16, β = 0.029, γ = 0.0, and intercept = −1.72), which are successfully applicable to DNA gyrase of Gram-positive/Gram-negative pathogens. Both methods offer accurate predictions of the binding free energies of NBTIs against *S. aureus* and *E. coli* DNA gyrase. We are confident that this integrated modeling approach could be valuable in the *de novo* design and optimization of efficient NBTIs for combating resistant bacterial pathogens.

## 1 Introduction

Increasing bacterial resistance is a global health concern, leading to ineffectiveness of antibiotics. The World Health Organization (WHO) regularly announces about the increasing number of bacteria-caused deaths as well as the financial burden associated with the unrestrained and uncontrolled use of antibiotics in treating bacterial resistance.^1^ According to the WHO, around 4.95 million deaths were associated with antimicrobial resistance in 2019, and 1.27 million of the deaths were attributed to it.^2^ Considering the current situation, it is estimated that by 2050, the number of human deaths caused by bacterial infections would exceed 10 million per year.^3^ Consequently, the discovery of novel antibacterial agents for combating bacterial resistance is of imperative importance.

Among the various antibacterial targets, bacterial type II topoisomerases, such as DNA gyrase and its paralogous equivalent topoisomerase IV (topoIV), proved to be well validated in treating bacterial infections. The main function of DNA gyrase enzyme is to maintain a correct spatial topology of the DNA molecule through introduction of negative supercoils, while topoIV is responsible for DNA decatenation activity during the recombination and replication processes. Structurally, both bacterial type II topoisomerases are heterotetrameric enzymes, with DNA gyrase consisting of two GyrA and two GyrB subunits (A_2_B_2_) and topoIV consisting of two ParC and two ParE subunits (C_2_E_2_). GyrA/ParC subunits are catalytic domains that are responsible for cleavage and religation of the DNA, while GyrB/ParE subunits are the ATPase domains that provide energy for the enzymatic reaction.^4,5^ Consequently, the perturbation of the correct spatial DNA topology by intercalating small ligand molecules between DNA base pairs that concomitantly bind to the enzyme prevents the essential bacterial processes, which, in turn, lead to bacterial cell death.

**Figure 1:**
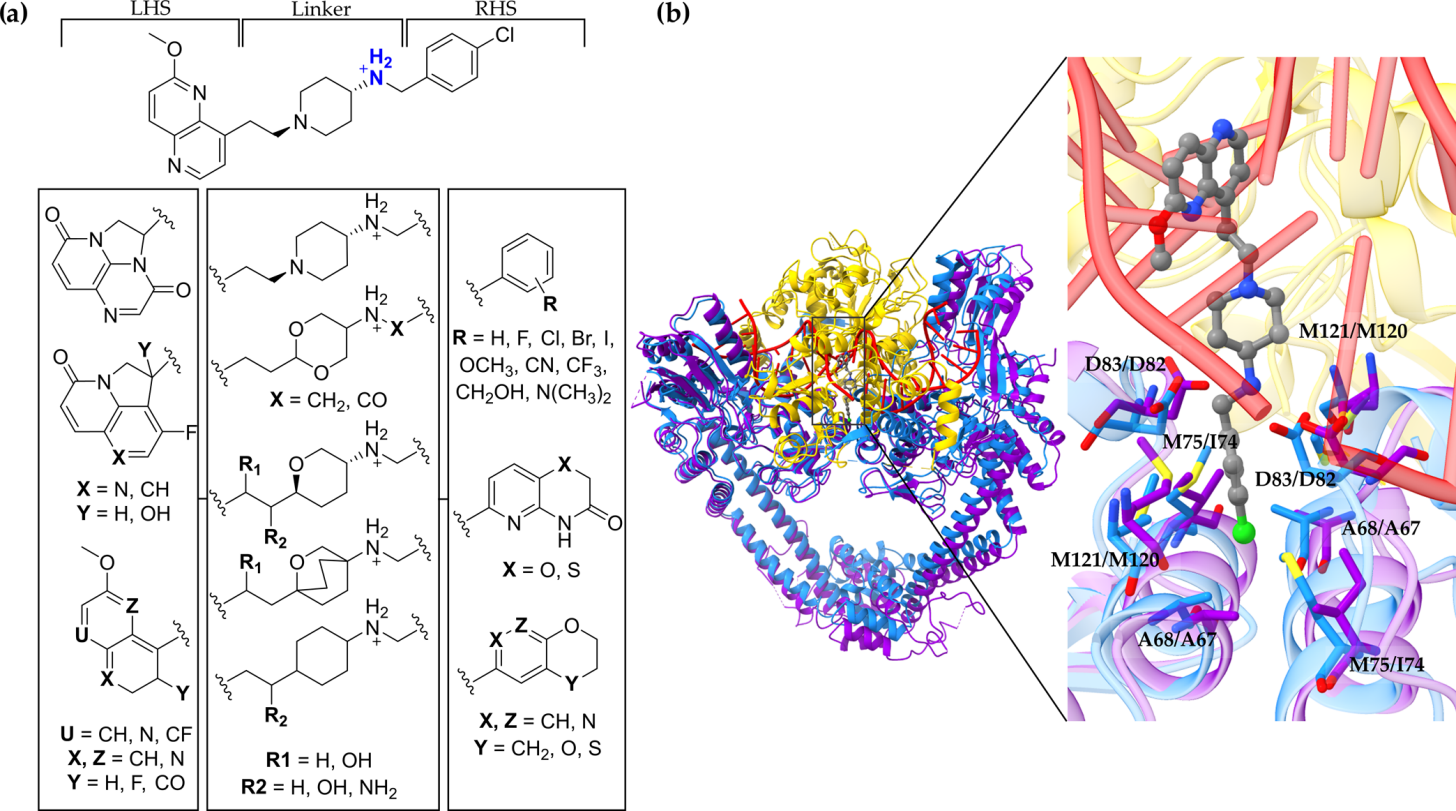
(a) Two-dimensional structure of an NBTI representative^12^ comprising the left-hand-side (LHS), linker, and right-hand-side (RHS) moieties and established structure–activity relationship (SAR) of the NBTIs used in this study. (b) Structural alignment of our recently disclosed *S. aureus* DNA gyrase (PDB ID: 6Z1A)^12^ and *E. coli* DNA gyrase (PDB ID: 6RKS)^18^ enzyme; *S. aureus* GyrA (cartoon representation in blue) and *E. coli* GyrA (cartoon representation in violet). The GyrB subunit is presented in cartoon representation in yellow and DNA in red. The amino acid residues important for binding of the NBTIs are in stick representation (for *S. aureus* GyrA: Ala68, Met75, Asp83, and Met121, i.e., for *E. coli* GyrA: Ala67, Ile74, Asp82, and Met120). The co-crystallized NBTI ligand (AMK12)^12^ is depicted as a ball-and-stick representation colored by heteroatoms.

A broadly known and commonly used class of intercalating antimicrobial agents that target these bacterial enzymes are fluoroquinolones.^6^ Some representatives of this class, including ciprofloxacin, levofloxacin, and moxifloxacin, are still used in the clinical practice, but unfortunately, their clinical use over several decades and frequent misapplication have promoted acquired resistance in bacteria.^6−8^ Approximately two decades ago, a new promising class of non-fluoroquinolone intercalating antibacterial agents commonly known as novel bacterial topoisomerase inhibitors (*alias* NBTIs) was disclosed (Figure 1a).^9−11^ These intercalating antibacterials inhibit the same bacterial type II topoisomerases as fluoroquinolones, however via a completely different inhibitory mechanism, i.e., stabilization of single-strand DNA breaks relative to the fluoroquinolones-induced stabilization of double-strand DNA breaks.^11,12^ Moreover, in contrast to fluoroquinolones, NBTIs bind to an alternative, close, but not overlapping binding site in bacterial topoisomerases, thereby largely overcoming cross-resistance with the fluoroquinolones (Figure 1b).^11−13^

The beginnings of the development of NBTIs as a new class of antibacterials reach back nearly 20 years ago, with the discovery of the first encouraging NBTI, viquidacin (NXL-101), that underwent phase I clinical trials. However, it was discontinued due to its hERG-related cardiotoxic issues manifested as prolonged QT signals in the heart.^14^ Further important breakthrough was the unveiling of the very first crystal structure of *Staphylococcus aureus* DNA gyrase enzyme in complex with an NBTI ligand (GSK299423)^11^ that enabled structure-based design/optimizations of numerous NBTI variants with improved antibacterial activity (Figure 1a). The most advanced NBTI is gepotidacin, which currently finishes the third phase of clinical trials for the treatment of uncomplicated urogenital gonorrhea^15,16^ and uncomplicated urinary tract infection commonly caused by *Escherichia coli*.^17^

As represented in Figure 1a, the NBTI antibacterials comprise three key parts: a heteroaromatic “left-hand-side” (LHS) that intercalates between central DNA base pairs, an aromatic/heteroaromatic “right-hand-side” (RHS) that binds into a deep, non-catalytic hydrophobic binding pocket formed at the interface of both GyrA/ParC subunits in DNA gyrase/topoIV, and a specific linker connecting them. It was found that the linker moiety is an essential NBTI structural determinant that ensures not only proper spatial orientation and conformation of the entire ligand for establishing key interactions with amino acid residues delineating GyrA/ParC subunits in DNA gyrase/topoIV but also suitable physicochemical properties of NBTIs.^19−21^ This imposed introduction of various linker variants, including aminopiperidine, tetrahydroindazole, oxabicyclooctane, tetrahydropyrane, dioxane, and cyclohexane (Figure 1a).^9,22−28^ The basic nitrogen on the linker moiety (Figure 1a, represented in blue) was found to be of exceptional importance for the NBTI’s binding affinity and consequently their antibacterial potency through establishing a key ionic interaction with GyrA Asp83, i.e., Asp82 residue of *S. aureus* and *E. coli* DNA gyrase.^10,11,19,24^ Moreover, the available X-ray and cryo-electron microscopy (cryo-EM) structural data of *S. aureus* DNA gyrase (PDB ID: 6Z1A)^12^ and *E. coli* DNA gyrase (PDB ID: 6RKS)^18^ in complex with DNA and intercalated NBTI ligands (AMK12 and gepotidacin, respectively) revealed a high level of conservancy of amino acid residues delineating NBTI binding sites in both enzymes of Gram-positive and Gram-negative bacteria (Figure 1b).^12,18−20^ This in turn enabled a more intuitive and rationally grounded design/optimization of potent NBTI antibacterials on a structure-based level.^12,27,29−33^ It should be stressed, however, that the majority of structure-based strategies (e.g., pharmacophore modeling and screening, molecular docking calculations) utilized for NBTI’s design/optimization provide a satisfactory insight into their binding mode that is usually quantified by a scoring function derived empirically.^34^ Indeed, these *in silico* methodologies are capable of relatively correct prediction of the NBTI binding mode; however, the accurate prediction of their binding affinity is commonly demanding. The latter one is of particular concern considering the expectations that docking-derived binding affinity of a ligand is indeed a good indicator of its actual binding affinity, which in turn results in selection of NBTI hit candidates that frequently fail at the later *in vitro* stages.^30^ Put differently, one should implement a set of more vigorous and accurate enough *in silico* modeling methodologies in describing the ligand’s binding and subsequent derivation of its binding affinity for the biological target under consideration.

In the present paper, we discuss the development and validation of multidimensional predictive binding site surrogate models of *S. aureus* and *E. coli* DNA gyrase enzymes. The models aimed at predicting enzyme inhibitory potencies of structurally diverse NBTI analogues with high accuracy as well as identification of the relevant amino acid residues for their binding and affinity in both bacterial targets. Those NBTI analogues with highly predicted binding affinities by both multidimensional binding site surrogates were selected and subsequently subjected to molecular dynamics (MD) simulations for profiling of their dynamic behavior and characterization of their ligand–protein interactions. In addition, the linear interaction energy (LIE) method was employed for prediction of binding free energies (Δ*G*_bind_pred_^°^) for selected NBTIs utilizing an in-house derived set of LIE weighting parameters (α, β, and γ). Moreover, one can recognize this integrated *in silico* modeling approach as an important advantage in the identification of *de novo* designed/optimized NBTI analogues with strong enzyme inhibitory potencies against DNA gyrase enzymes of Gram-positive and Gram-negative bacterial pathogens, which nowadays are of considerable significance in combating bacterial resistance.

## 2 Results and Discussion

### 2.1 Multidimensional QSAR Simulations

The multidimensional QSAR simulations were based on a family of 200 parent models for each model separately (*S. aureus* and *E. coli* DNA gyrase) that differ in the quasi-atomistic properties mapped on their pseudosurface. During the modeling, the family of each DNA gyrase surrogate model evolved for a different number of crossover cycles that correspond to a different number of generations (see Table 1).

**Table 1:** Summary of the Multidimensional QSAR Models of the DNA Gyrase Enzyme Originating from *S. aureus* and *E. coli* Obtained by the Quasar^X^ Partial Least-Squares Genetic Algorithm (PLS-GA) Method^a^

model	number of crossovers	number of generations	*r*^2^	*q*^2^	rmsd training	max. training	*p*^2^	rmsd test	max. test
*S. aureus* DNA gyrase	34,000	170	0.795	0.791	1.5	14.9	0.756	1.5	5.1
*E. coli* DNA gyrase	75,000	375	0.810	0.806	1.3	8.4	0.582	1.5	4.3

a *r*^2^: Pearson’s correlation coefficient, *q*^2^: cross-validated *r*^2^, and *p*^2^: predictive *r*^2^ for the test set; the rmsd and maximal deviation from the experimental binding affinity are given as a factor (off) in IC_50_.

The multidimensional *S. aureus* DNA gyrase QSAR model was derived on 160 NBTI_SA_ training compounds (Table 1 and Supporting Information, Figure S1a). The model converged at a cross-validated *r*^2^ value (*q*^2^ = 0.791, at 34000 crossovers), and its predictive performance was evaluated on 39 test ligands (*p*^2^ = 0.756, predictive *r*^2^). The calculated binding affinity of the training and test ligands differed on average by a factor of 1.5 from the experimental values, while the maximum deviation of a single ligand was 14.9 for the training set and 5.1 for the test set. In a similar manner, the *E. coli* DNA gyrase multidimensional QSAR model grounded on 108 NBTI_EC_ training compounds (Table 1 and Supporting Information, Figure S1b) reached a cross-validated *r*^2^ value *(q*^2^ = 0.806, at 75000 crossovers), which the predictive power was evaluated on 25 test ligands (*p*^2^ = 0.582, predictive *r*^2^). The average calculated binding affinity of the training and test ligands deviated from their experimental values by a factor of 1.3 and 1.5, respectively, whereas the maximum deviation of a single compound resembles a value of 8.4 for the training set and 4.3 for the test set. In addition, the sensitivity of both models to the biological data used (IC_50_) was assessed by conducting a series of 20 scramble (*Y*-randomization) trials per model (Supporting Information, Table S1). As demonstrated, one can perceive that the resulting predictive *p*^2^ values for both models are significantly lower relative to those obtained by the selected models (Table 1); these results clearly pinpoint that the selected multidimensional models for both enzymes are indeed sensitive to the biological data employed and consequently can be used for the prediction of binding affinities of newly designed/optimized NBTIs.

**Figure 2:**
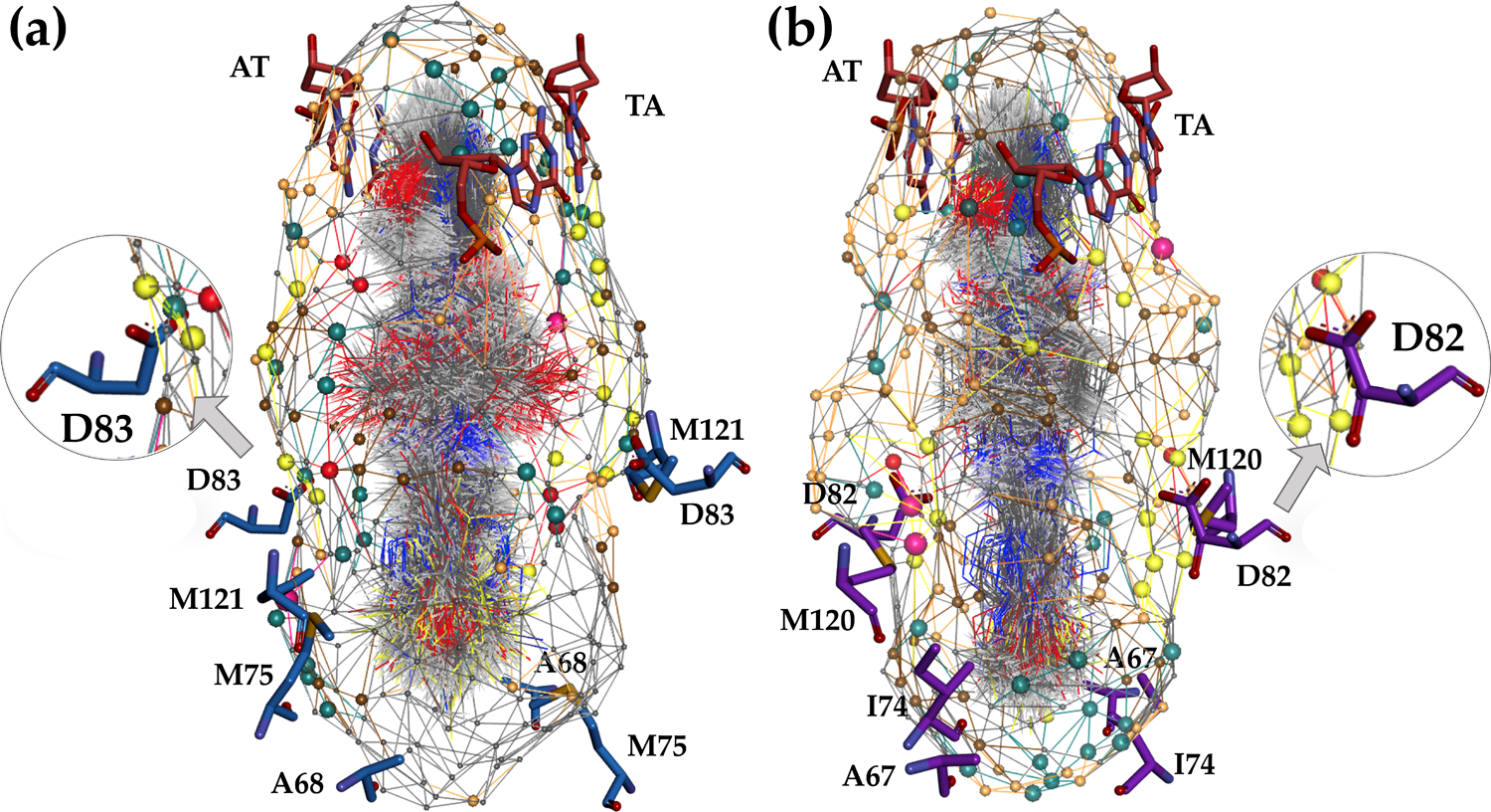
Multidimensional quasi-atomistic binding-site surrogate models of (a) *S. aureus* DNA gyrase enzyme and (b) *E. coli* DNA gyrase enzyme. For clarity and easier interpretation of the quasi-atomistic properties mapped on the pseudosurface of the models, the corresponding key amino acid residues crucial for NBTI binding and affinity (e.g., A68, M75, D83, and M121 from the *S. aureus* DNA gyrase–stick representation, inset: blue, i.e., A67, I74, D82, and M120 from the *E. coli* DNA gyrase–stick representation, inset: violet) as well as the central DNA base pairs [e.g., adenine-thymine (AT-TA) base pairs, stick representation, inset: red] were artificially inserted utilizing the available experimental data.^12,18^

By comparing the available crystal structure of *S. aureus* DNA gyrase in complex with the AMK12 ligand, it can easily be recognized that the *S. aureus* DNA gyrase model’s quasi-atomistic properties mapped on its pseudosurface are correctly reproducing some of the key amino acid functionalities for NBTI binding and affinity [e.g., the yellow hydrogen-bonding (HB) acceptor particles that correspond to the GyrA Asp83 carboxylate moiety as depicted in Figure 2a]. In addition, the hydrophobic features (brown and gray particles) cover a significant part of the pseudosurface and correctly reflect the hydrophobicity of the binding pocket delineated by Ala68, Met75, and Met121 residues.

Considering the structural resemblance that both enzymes share, similar arrangement of quasi-atomistic properties can also be noticed on the pseudosurface representing the *E. coli* DNA gyrase surrogate model (Figure 2b), i.e., hydrogen-bonding acceptor particles (yellow spheres) that correspond to the GyrA Asp82 carboxylate group as well as the hydrophobic entities (brown and gray particles) that coincide with Ala67, Ile74, and Met120 residues delineating the NBTI binding pocket in *E. coli* DNA gyrase.^18^

Thus, validated, the constructed quasi-atomistic *S. aureus* and *E. coli* DNA gyrase surrogate models were further challenged for prediction of the binding affinities for 26 structurally optimized NBTIs (**L01**–**L26**) selected from our recent studies with experimentally determined inhibitory potencies, which were compiled as an external, independent set not used in the development of the models (Table 2).^26,32^ Since the chemical property domain of the NBTIs encompassing the external set corresponds to the property space of the models (similar structural functionalities as the training set compounds) and their activity range is within the broader range of activities of the training set molecules, one could expect reliable predictions of their binding affinities instead of their extrapolation.

**Table 2:**
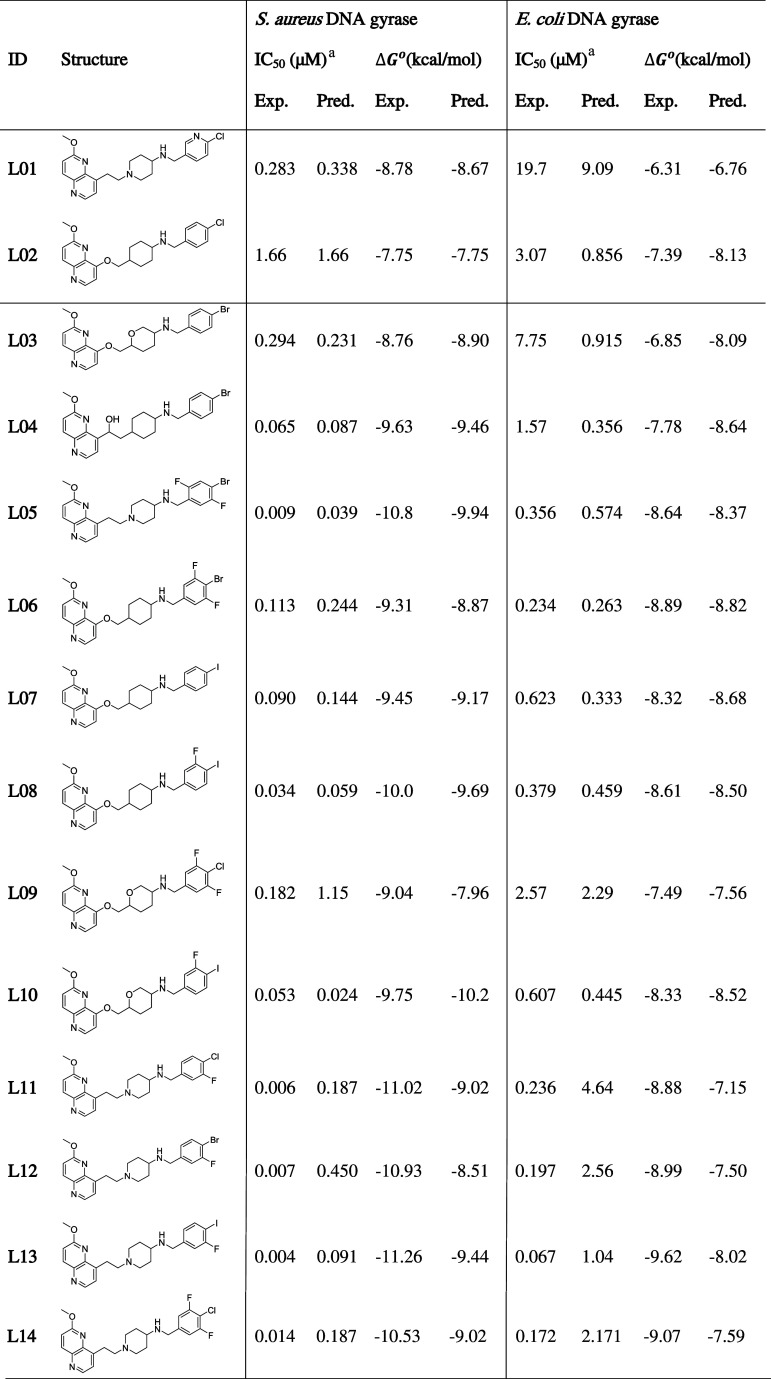

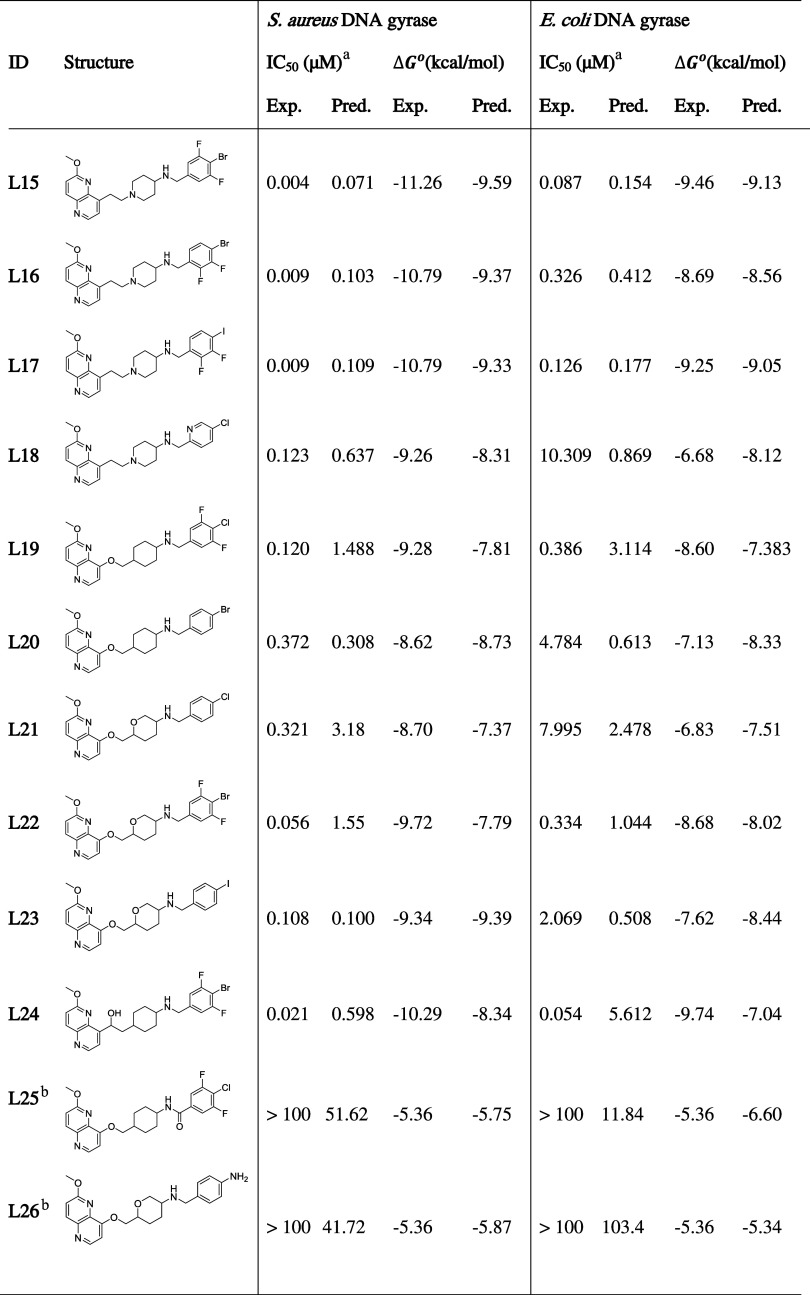
External, Independent Set of 26 NBTI Antibacterials with Experimentally Evaluated Inhibitory Potencies against *S. aureus*, i.e., *E. coli* DNA Gyrase Enzyme (IC_50_), Selected from Our Previous Studies for Additional External Validation (Prediction of Binding Affinities) of the Constructed Multidimensional QSAR Models

a The experimental IC_50_ values for *S. aureus* and *E. coli* DNA gyrase are available in our recent publications.^26,32^

b NBTI ligands used as negative controls; the experimental IC_50_ values are available in our recent publications.^35,36^

**Figure 3:**
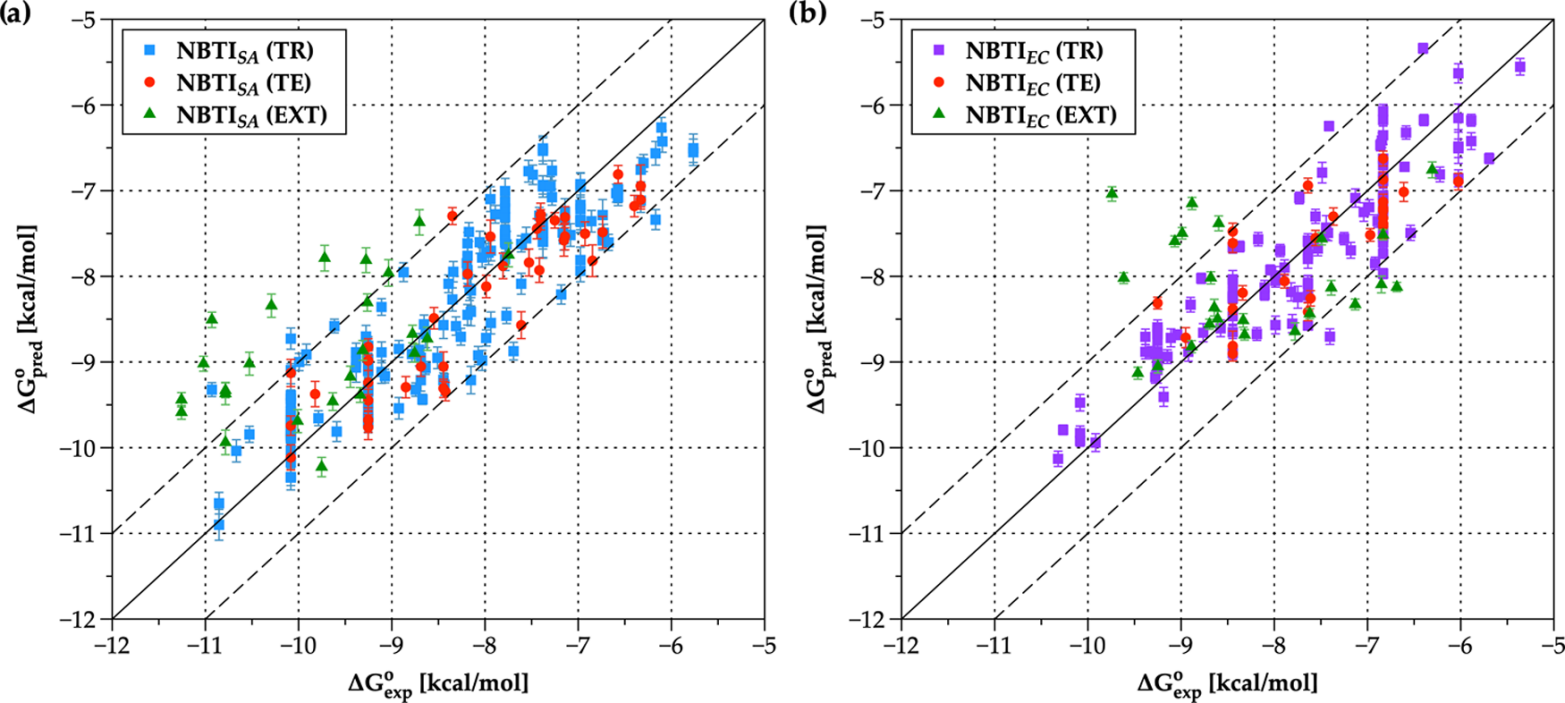
Two-dimensional plots representing the correlation of experimental (Δ*G*_exp_^o^) versus predicted (Δ*G*_pred_^o^) binding affinities in kcal/mol of NBTIs employed in this study (NBTI_SA_ and NBTI_EC_, respectively) as derived by (a) *S. aureus* DNA gyrase multidimensional surrogate model (the training set objects are depicted as solid blue squares), and (b) *E. coli* DNA gyrase multidimensional surrogate model (the training set objects are depicted as solid violet squares). For both models, the test set compounds are represented as solid red circles, while the external set compounds are represented as solid green triangles. The error bars correspond to the cumulative standard deviation (SD) values calculated for over 200 parent models.

As demonstrated in Table 2 and Figure 3, it is apparent that the predictive power of the *S. aureus* DNA gyrase model outperforms the *E. coli* DNA gyrase model to a certain extent. This slight discrepancy is most probably a consequence of the quality of four-dimensional (4D) representation of the ligand’s ensembles obtained by the flexible docking of the ligands that is directly related to the quality of the experimental structural data utilized (e.g., 2.3 Å resolution of *S. aureus* DNA gyrase X-ray structure^12^ relative to the 4.0 Å resolution of *E. coli* DNA gyrase cryo-EM structure^18^). It is evident that both models relatively accurately predict the binding affinities of NBTIs containing oxymethylene cyclohexane (e.g., **L06**–**L08**) and oxymethylene tetrahydropyran linkers (e.g., **L09**, **L10**, and **L22**).

**Figure 4:**
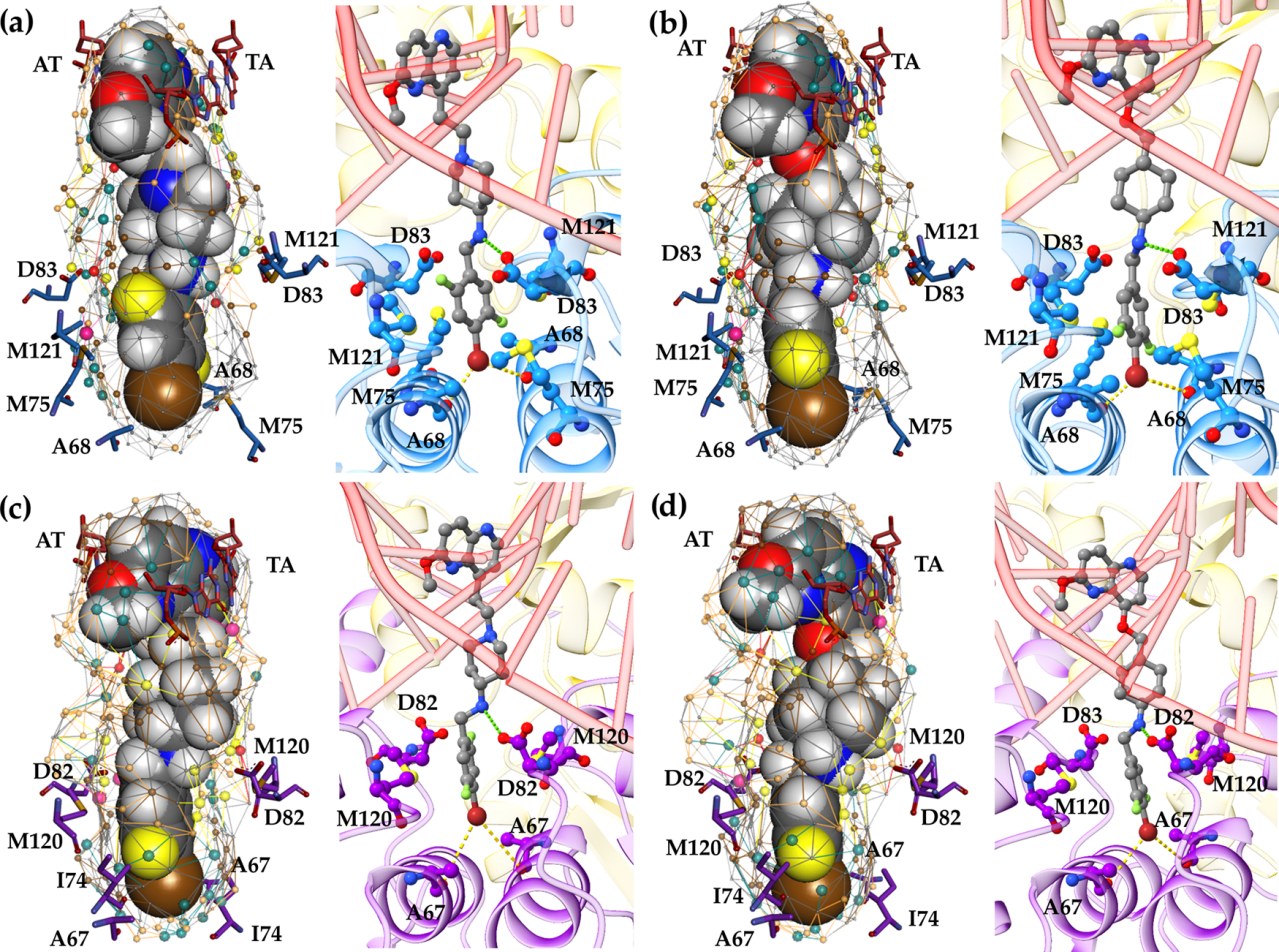
Comparison of Quasar^X^ predicted and docking-derived binding poses of compounds **L05** and **L06** in *S. aureus* DNA gyrase (inset: blue) and *E. coli* DNA gyrase (inset: violet). (a, b) Binding conformations of **L05** and **L06** in the *S. aureus* DNA gyrase model and (c, d) binding conformations of **L05** and **L06** in the *E. coli* DNA gyrase model. The mapped yellow quasi-atomistic properties (hydrogen-bonding acceptors) correspond to the carboxyl oxygen atoms of Asp83/Asp82 residues of *S. aureus*, i.e., *E. coli* GyrA subunits, which are crucial for establishing strong NBTI–enzyme ionic interactions (green dots). GyrA subunits are represented as a cartoon, and the ionic interactions between ligand’s protonated nitrogen and the aspartate residues are shown as green dots, while the bifurcated halogen-bonding interactions between RHS’s bromine atom and backbone carbonyl oxygens of Ala68/Ala67 residues are depicted as yellow dots.

However, it is interesting to note that binding affinities, in particular those of compounds **L01**, **L11**, **L12**, **L14**, and **L18**, are significantly poorly predicted by the *E. coli* DNA gyrase model compared to those obtained by the *S. aureus* DNA gyrase model. Although these NBTIs encompass an aminopiperidine-naphthyridine moiety that is comprehensively covered by the training set ligands (the models), they contain a variety of RHS fragments (e.g., chloropyridine, mono- and difluoro *p*-halogenated phenyl moieties), which are outside the structural space of the models, and expectedly, their binding affinities are poorly derived. The same also stands for the modest predictions of some of these compounds by the *S. aureus* DNA gyrase model (e.g., **L01**, **L11**, **L12**, and **L14**). Despite the structural differences of RHS fragments, it is also notable that some compounds, in particular **L05** (an naphthyridine analogue) and **L06** (oxymethylene cyclohexane derivative), are well predicted by both models (Table 2), which results are in line with their calculated binding conformations, as well (Figure 4). Put differently, both compounds contain difluorophenyl RHS moieties that differ in the position of the fluorine atoms, which significantly affects the enzyme’s inhibition. While **L06** is equally potent in both enzymes, **L05** is 30-fold stronger against *S. aureus* DNA gyrase (Table 2).

Moreover, both multidimensional DNA gyrase surrogate models also relatively accurately predicted the binding affinity of compounds **L25** and **L26**, which served as a negative control (IC_50_ > 100 μM) for additional validation of their predictive performance (Table 2). It is interesting to note that these NBTIs comprise structural features that are not covered in the training sets of both models (e.g., **L25**—an amide containing NBTI and **L26**—an NBTI with a *p*-aminophenyl RHS moiety),^35,36^ yet their predicted relative binding free energies differ around ±1.0 kcal/mol on average (Table 2).

Nevertheless, considering the structural diversity of these NBTI analogues as well as their reasonably predicted binding affinities as derived by the multidimensional QSAR models, we selected 10 compounds (**L01**–**L10**) for further assessment of their dynamic behavior and prediction of binding affinities by MD simulations.

### 2.2 Molecular Dynamics Simulations

**Figure 5:**
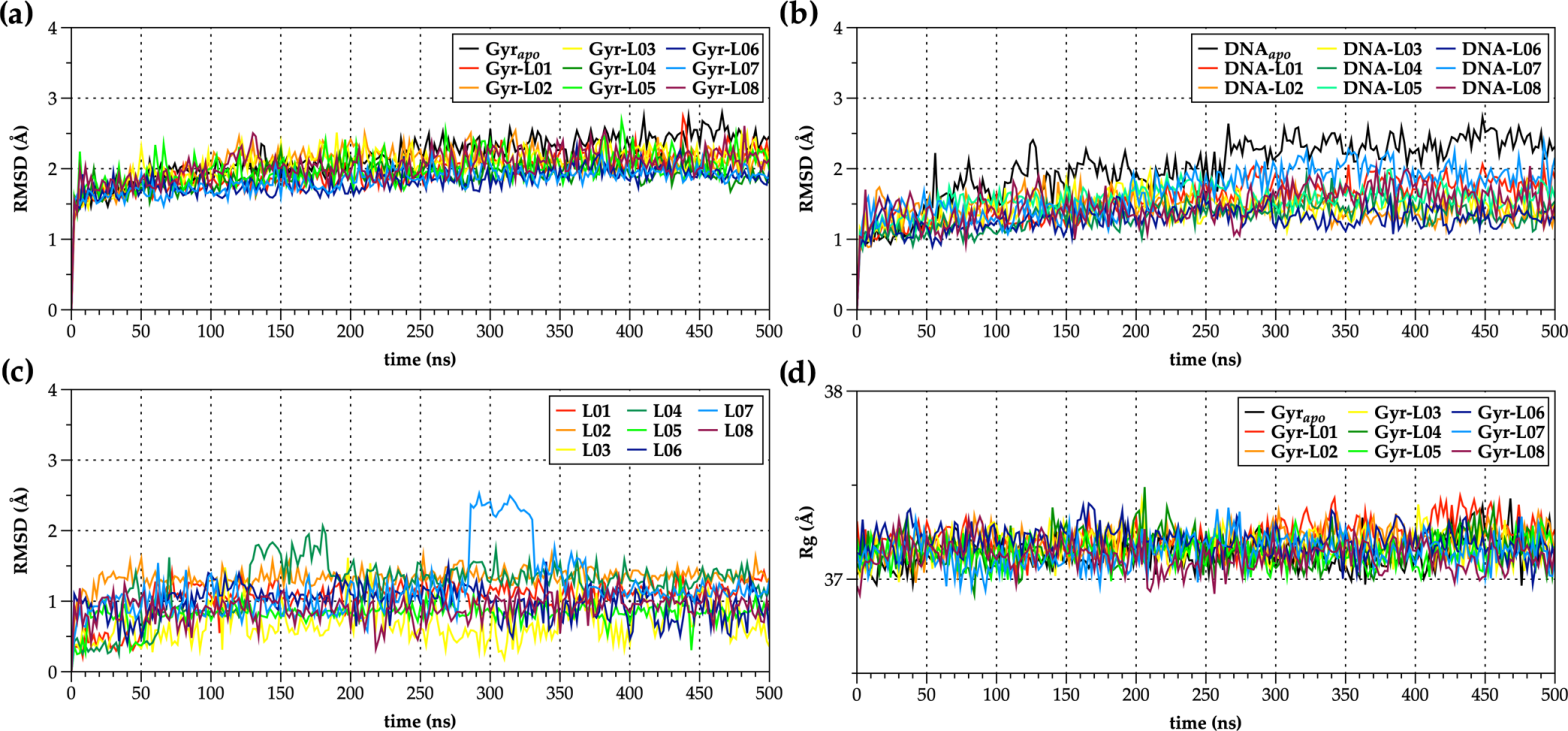
Root-mean-square deviation (RMSD [Å]) and radius of gyration (*R*_g_ [Å]) plots of 500 ns MD simulations for the ligand-free (Gyr_apo_) *S. aureus* DNA gyrase enzyme (PDB ID: 6Z1A)^12^ and its NBTI-ligated complexes (Gyr-**L01**–**L08**). (a) Backbone protein RMSDs; (b) DNA RMSDs; (c) ligand RMSDs (**L01**–**L08**); and (d) protein *R*_g_ plots of the ligand-free (Gyr_apo_) and ligated systems (Gyr-**L01**–**L08**).

**Figure 6:**
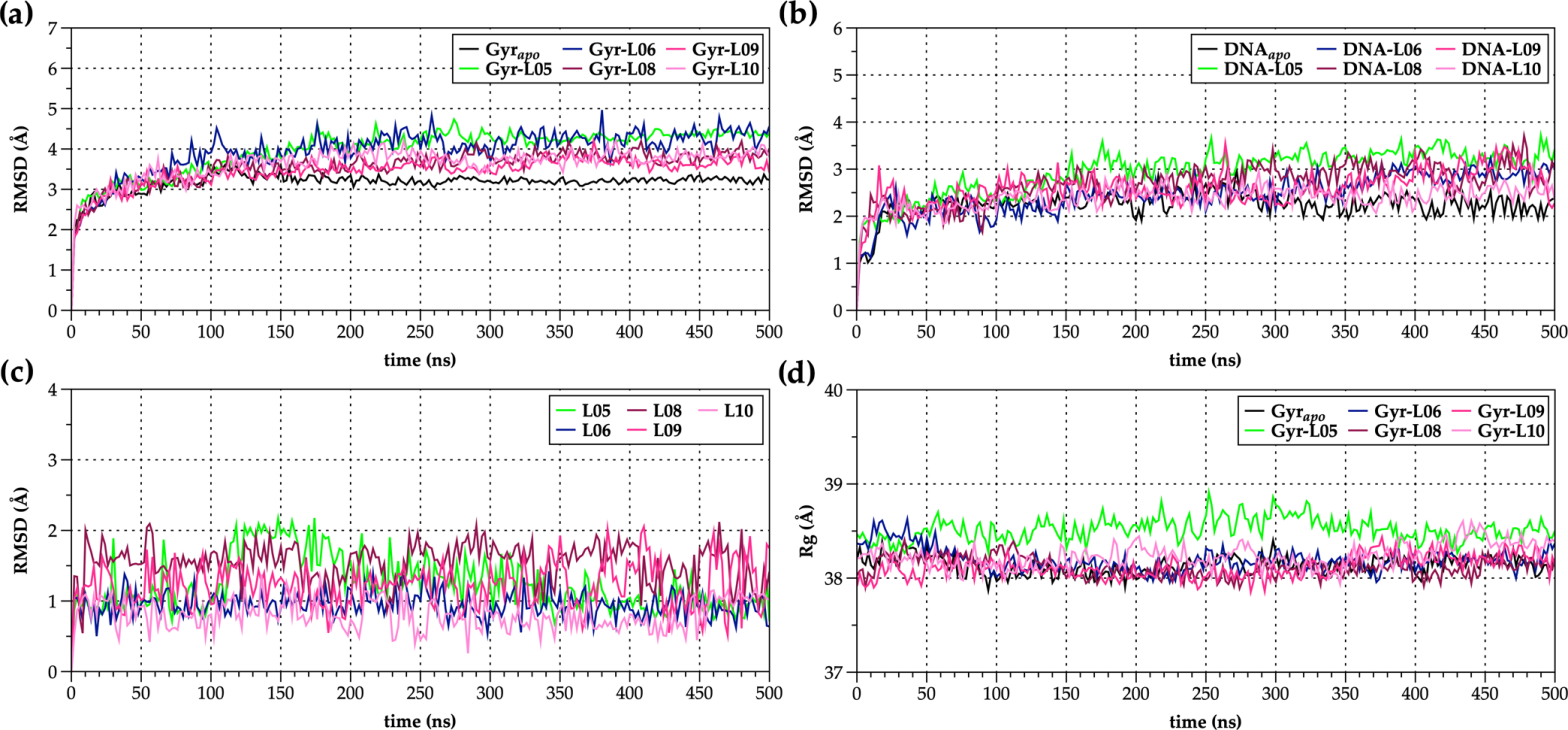
Root-mean-square deviation (RMSD [Å]) and radius of gyration (*R*_g_ [Å]) plots of 500 ns MD simulations for the ligand-free (Gyr_apo_) *E. coli* DNA gyrase enzyme (PDB ID: 6RKS)^18^ and its NBTI-ligated complexes (Gyr-**L05**, Gyr-**L06**, and Gyr-**L08**–**L10**). (a) Backbone protein RMSDs; (b) DNA RMSDs; (c) ligand RMSDs (**L05**, **L06**, and **L08**–**L10**); and (d) protein *R*_g_ plots of the ligand-free (Gyr_apo_) and ligated systems (Gyr-**L05**, Gyr-**L06**, and Gyr-**L08**–**L10**).

To investigate the dynamic profile and binding of the selected NBTI compounds (**L01**–**L10**) to *S. aureus* and *E. coli* DNA gyrase enzymes, the compounds underwent 500 ns molecular dynamics (MD) simulations. The MD simulations were performed for eight *S. aureus* DNA gyrase (PDB ID: 6Z1A)^12^ complexes assembled by utilizing **L01**–**L08** previously derived docked conformations, i.e., five *E. coli* DNA gyrase (PDB ID: 6RKS)^18^ complexes with **L05**, **L06**, and **L08**–**L10** ligands (Table 2). Moreover, for comparison purposes, MD simulations on *apo* (ligand-free; Gyr_apo_) forms of both enzymes (*S. aureus* and *E. coli* DNA gyrase) were conducted, as well. The resulting MD production trajectories of each investigated system (*S. aureus* and *E. coli* DNA gyrase) were in the first instance analyzed to appraise their stability, the dynamics profile, and the compactness by monitoring their root-mean-square deviation (RMSD) and radius of gyration (*R*_g_) (Figures 5 and 6). Considering the complexity of investigated systems, RMSD plots of each system’s entities (e.g., backbone protein, DNA, and NBTI ligands) over the entire simulation time were calculated.

Figures 5a and 6a show the backbone protein RMSDs of the *apo* (ligand-free; Gyr_apo_) and NBTI-ligated *S. aureus* DNA gyrase (Gyr-**L01**–**L08**), i.e., *E. coli* DNA gyrase (Gyr-**L05**, Gyr-**L06**, and Gyr-**L08**–**L10**) systems, respectively. As demonstrated in Figure 5a, there are no significant backbone protein RMSD deviations between the *apo* and *S. aureus* DNA gyrase NBTI complexes (∼1.5–2.8 Å) that indicate well-equilibrated, stable systems over the entire simulation time.

A similar outcome can also be observed for *E. coli* DNA gyrase systems, i.e., backbone protein RMSDs deviate ∼3.0–4.0 Å for most of the investigated complexes with slight fluctuations for the Gyr-**L05** and Gyr-**L06** complexes (Figure 6a). In contrast to the enzyme, *S. aureus* DNA RMSD is slightly higher for the *apo* (ligand-free) form (∼1.0–2.8 Å) relative to those of complexated systems (∼1.0–2.0 Å), indicating stabilization of the DNA molecule by intercalation of the NBTI’s LHS moieties between central DNA base pairs (Figure 5b). However, in *E. coli*, there are no substantial DNA fluctuations between the *apo* and complex systems, in which RMSD values differ by ∼0.5 Å (Figure 6b).

Among the investigated NBTIs (Figures 5c and 6c), one can observe a relatively good stability for the majority of them within *S. aureus*, i.e., *E. coli* DNA gyrase binding site, except for **L04** and **L07** ligands in *S. aureus* DNA gyrase, i.e., **L05** and **L08** ligands in *E. coli* DNA gyrase, in which occasional RMSD fluctuations appear as a consequence of the axial rotation of their RHS moieties within the enzyme’s binding sites. These findings are in agreement with the evaluations of the compactness of the systems, as represented by the calculated *R*_g_ plots (Figures 5d and 6d).

### 2.3 Ligand–Protein Interactions

#### 2.3.1 Hydrogen-Bonding and Hydrophobic Contact Analysis

**Figure 7:**
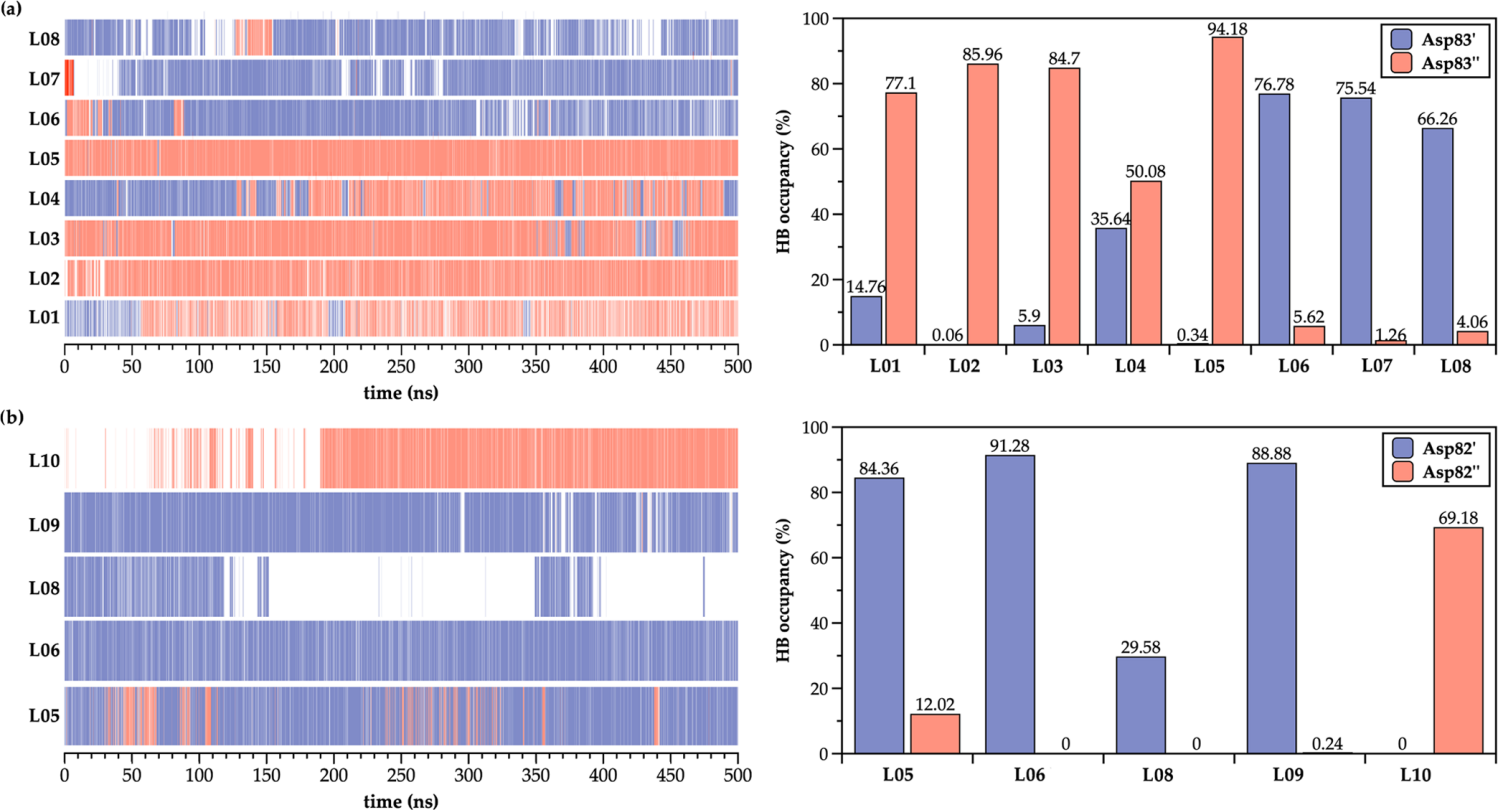
Hydrogen-bonding occupancy analysis of 500 ns MD-simulated NBTI compounds (**L01**–**L10**) with (a) GyrA Asp83′/Asp83″ residues of *S. aureus* DNA gyrase and (b) GyrA Asp82′/Asp82″ residues of *E. coli* DNA gyrase. Only direct ionic interactions between ligands and GyrA aspartate residues were considered.

The hydrogen-bonding (HB) occupancy analysis of the investigated NBTIs with amino acid residues covering the NBTI’s binding site in *S. aureus* and *E. coli* DNA gyrase enzymes shows that almost all of them interact mainly with Asp83 (Figure 7a), i.e., Asp82 residue (Figure 7b) from a sole GyrA subunit in both enzymes during the entire simulation time. Such an outcome was expected to a certain extent considering the predominantly hydrophobic nature of the NBTI binding site in bacterial topoisomerases. The only exception is the compound **L04** in *S. aureus* DNA gyrase, which establishes almost balanced direct HB interactions with Asp83′ (35.64%) and Asp83″ (50.08%) residues from each GyrA subunit. Such a balanced HB interaction is most probably due to the free axial rotation of the amino-cyclohexane linker moiety; this finding is congruent with the experimental evidence that a direct ionic interaction(s) between the linker’s protonated nitrogen and GyrA aspartate residue is crucial for the NBTI antibacterial potency (Table 2 and Figure 7a).^11,18^

The comparison of HB occupancy for NBTI ligands (e.g., **L05**, **L06**, and **L08**) active against both *S. aureus* and *E. coli* DNA gyrase enzymes (Table 2) shows that these compounds establish relatively strong ionic interactions over the entire simulation time with only a single GyrA Asp83, i.e., Asp82 residue. These steady ionic interactions (∼2.52–3.40 Å for *S. aureus* DNA gyrase, i.e., ∼2.45–2.67 Å for *E. coli* DNA gyrase) are undoubtedly one of the key interacting elements accounting for the stability of these ligands within the enzyme binding pockets, which in turn is notably reflected on their relatively strong inhibitory potencies in both enzymes (Table 2).^18^

**Figure 8:**
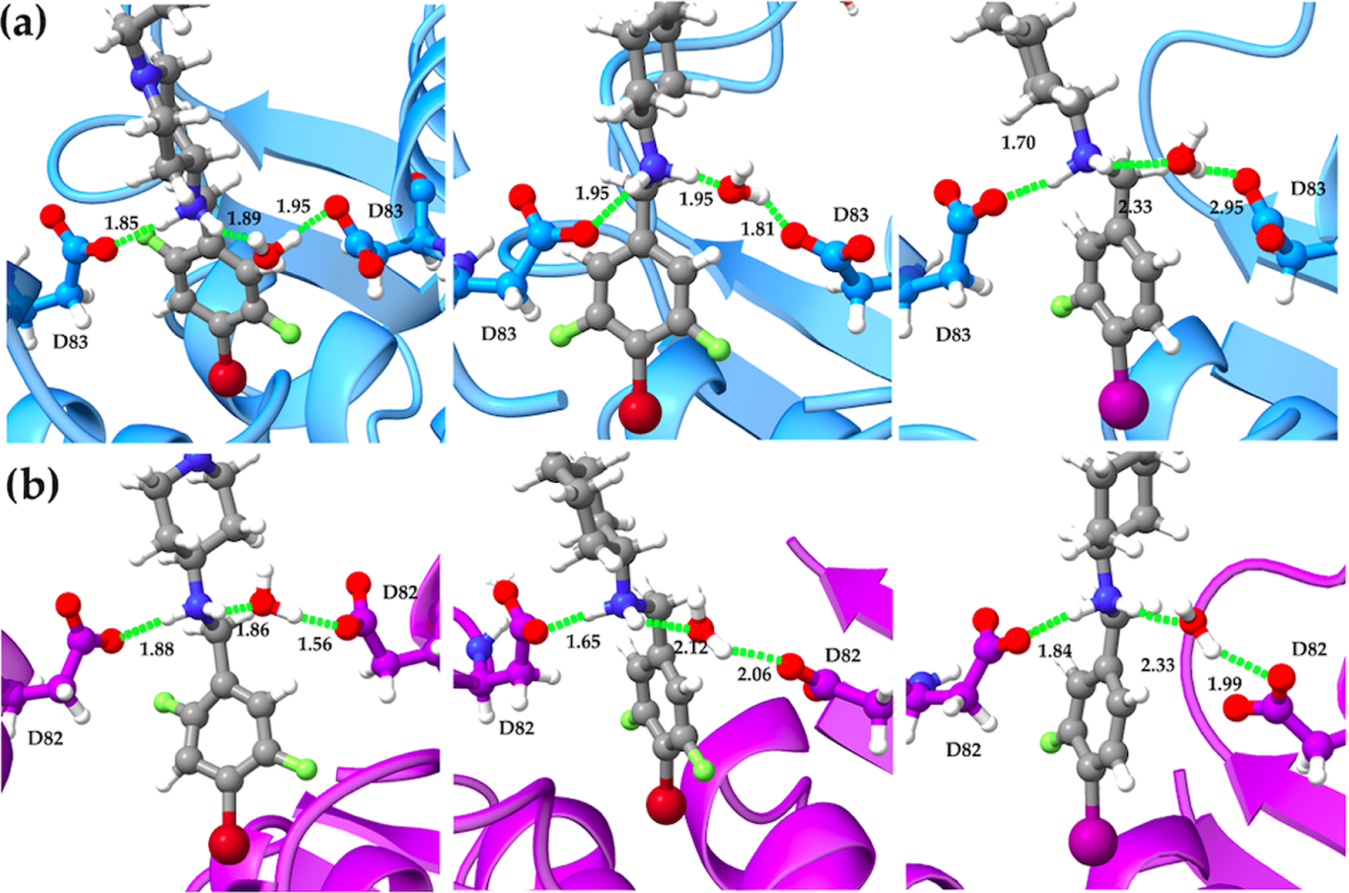
NBTI direct ionic interactions between the Asp83/Asp82 residue from one GyrA subunit and ligand’s protonated nitrogen as well as indirect water-mediated HB interactions with the Asp83/Asp82 residue from the second GyrA subunit. (a) *S. aureus* DNA gyrase (left **L05**, middle **L06**, and right **L08**). (b) *E. coli* DNA gyrase (left **L05**, middle **L06**, and right **L08**). The GyrA subunits are represented as cartoon (*S. aureus* GyrA in blue and *E. coli* GyrA in violet), while NBTI ligands (gray) are shown as ball and stick representation and colored by heteroatoms. The direct and water-mediated ionic interactions between the ligand’s protonated nitrogen and the aspartate residues from both GyrA subunits are depicted as green dots.

It should be stressed, however, that relative to other amino acid residues (e.g., Ala68, Gly72, Met75, and Met121 in *S. aureus* DNA gyrase, i.e, Ala67, Gly71, Met74, and Met120 in *E. coli* DNA gyrase), which delineate the deep hydrophobic binding pocket of NBTIs in DNA gyrase enzymes, the GyrA aspartate residues (e.g., Asp83, i.e., Asp82 in *S. aureus* and *E. coli* DNA gyrase) are solvent-exposed as demonstrated by the calculated solvent-accessible surface area (SASA) plots (Supporting Information, Figure S2). Put differently, the analysis of MD production trajectories of the investigated NBTIs (**L01**–**L10**) revealed that while the NBTI’s linker basic nitrogen establishes a direct ionic interaction with the aspartate residue from one GyrA subunit (Figure 7), it is also forming an indirect HB interaction with the aspartate residue from the second GyrA subunit through the surrounding water molecules in both enzymes (Figure 8); these findings coincide with the recent crystallographic experimental evidence.^13^

It is worth mentioning that these structural aspects are nicely emulated at the multidimensional pseudoatomistic *S. aureus* and *E. coli* DNA gyrase surrogate models, as well (Figure 2). Put differently, aside from the NBTI’s direct ionic interactions with one GyrA Asp83/Asp82 residue that are well defined by the HB acceptor features (yellow particles) as described previously, their indirect water-bridged HB interactions with the second GyrA Asp83/Asp82 residue are appropriately mimicked by the HB donor features (green particles) situated at the opposite side on the model pseudosurface (Figure 2).

**Figure 9:**
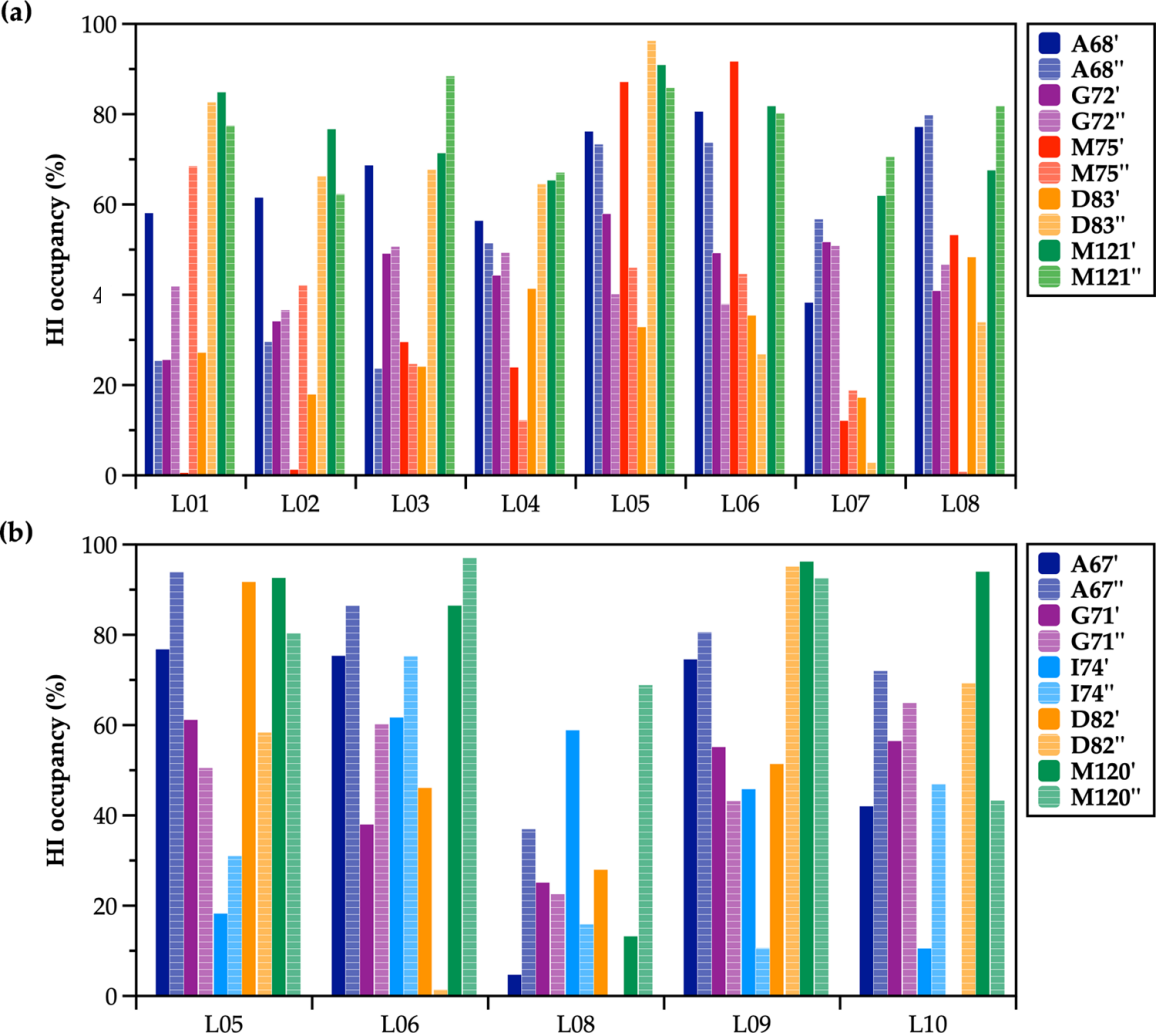
Hydrophobic interactions (HI) occupancy analysis of 500 ns MD-simulated NBTI compounds (**L01**–**L10**) with (a) GyrA Ala68, Gly72, Met75, Asp83, and Met121 residues of *S. aureus* DNA gyrase and (b) GyrA Ala67, Gly71, Ile74, Asp82, and Met120 residues of *E. coli* DNA gyrase. Solid bars correspond to binding site residues of one GyrA subunit, while striped bars represent those of the other GyrA subunit. An average distance of ≤4.0 Å was considered as a cutoff for measuring the hydrophobic contacts (e.g., aliphatic–aromatic carbons, aromatic–aliphatic carbons, aliphatic–aliphatic carbons, and carbon–halogen).

It should be pinpointed, however, that although these HB interactions are significantly contributing to the excellent antibacterial properties of NBTIs, their overall stability and antibacterial potency against the DNA gyrase enzyme is additionally enhanced by establishing a network of hydrophobic interactions (HI) between the NBTI’s RHS moiety and amino acid residues outlining the NBTI binding pocket (Figure 1). The HI occupancy analysis of MD-simulated NBTIs (**L01**–**L10**) revealed that Ala68/Ala67, Asp83/Asp82, and Met121/Met120 residues from both GyrA subunits in *S. aureus* and *E. coli* DNA gyrase enzymes almost equally contribute in forming the HI network over the entire simulation time, however, not with equal frequency (Figure 9). These amino acid residues are well conserved among DNA gyrase enzymes originating from various bacterial species. Nonetheless, it should be emphasized that even a negligible variation in a single amino acid residue could have a tremendous impact on the NBTI’s antibacterial potency between bacterial species. This is well demonstrated by the differences in the antibacterial potencies of compounds **L05**, **L06**, and **L08** making HI with Met75, i.e., Ile74 residue in *S. aureus* and *E. coli* DNA gyrase. Indeed, **L05** establishes a somewhat stable HI with the Met75 residue(s) in *S. aureus* DNA gyrase (Figure 9a) and only a few HI with the Ile74 residue(s) in *E. coli* GyrA (Figure 9b), which in turn might be the reason for its lower inhibition of *E. coli* DNA gyrase (Table 2). In contrast, the HI occupancy that **L06** establishes with Ile74 in *E. coli* DNA gyrase is high, which is also reflected in the stronger inhibition compared to compound **L05**. Moreover, the HI occupancy that **L08** provides is substantially lower in *E. coli* DNA gyrase relative to *S. aureus* DNA gyrase, which is reflected in a 10-fold lower inhibition of *E. coli* DNA gyrase by this NBTI ligand (Table 2).

### 2.4 Linear Interaction Energy (LIE)

The prediction of binding free energy (Δ*G*_bind_pred_^°^) for selected external NBTI ligands (Table 2, **L01**–**L10**) was conducted utilizing the linear interaction energy (LIE) method.^37^ Since default weighting parameters α = 0.5, β = 0.16, and γ = 0 were not applicable to our systems, suitable LIE parameters were determined from the resulting MD production trajectories obtained by MD simulations of 18 *S. aureus* DNA gyrase–NBTI complex systems comprising structurally diverse NBTIs (**A01**–**A18**) selected from the NBTI_SA_ library (Supporting Information, Table S2 and Figure S3). The adjustment of LIE weighting parameters was accomplished on 14 training systems (Supporting Information, Table S3; **A01**–**A05**, **A07**–**A09**, **A13**–**A18**) by means of linear fitting of their experimental binding free energies (Δ*G*_bind_exp_) and subsequently validated by predicting the binding free energies (Δ*G*_bind_pred_^°^) of four test systems not used in the parameter calibration procedure (Supporting Information, Table S3; **A6**, **A10**–**A12**).

**Figure 10:**
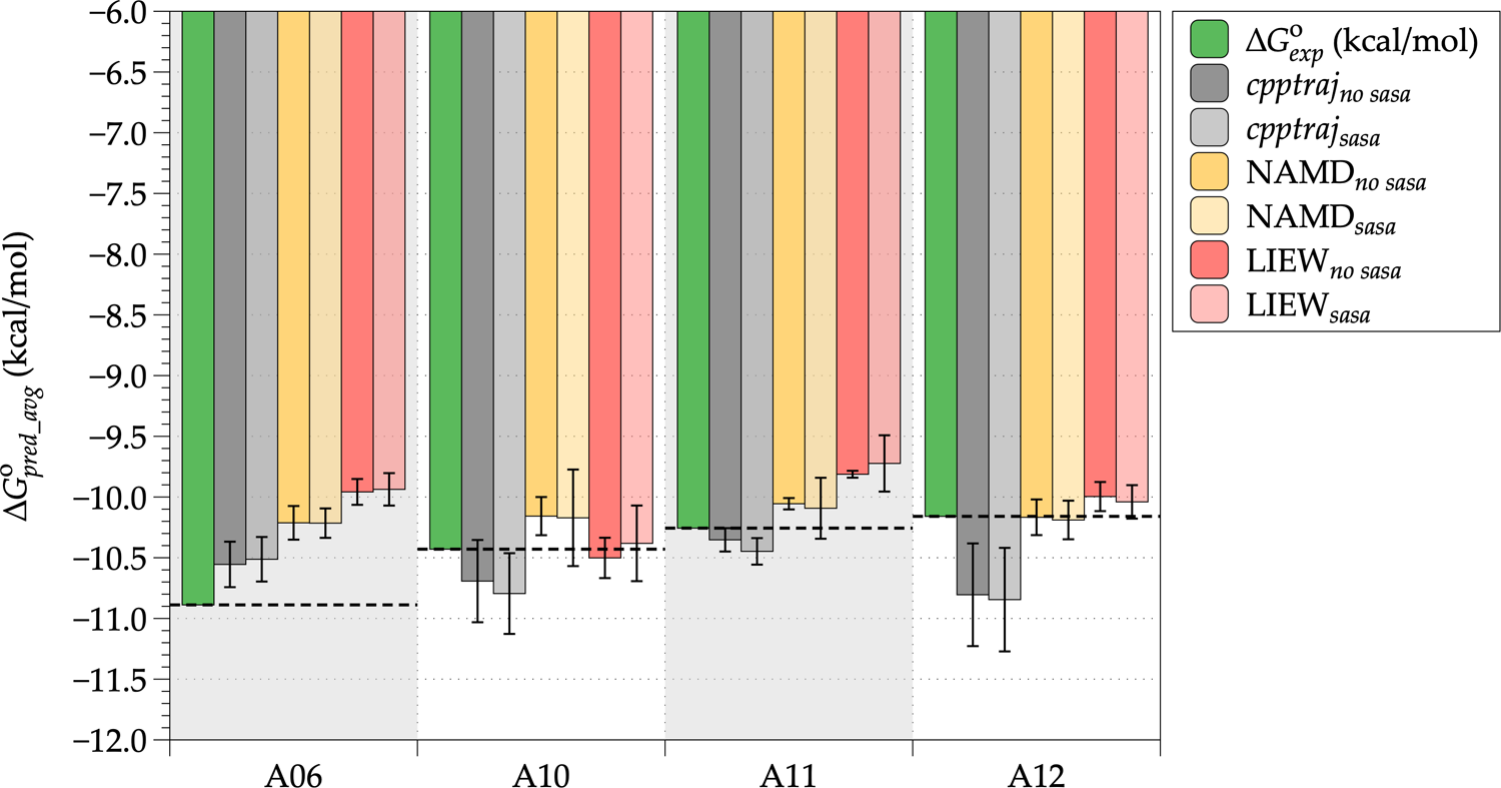
Comparison of the different calculation methods employed for deriving the relative binding free energies (Δ*G*_bind_pred_^°^) of the test set compounds (**A06**, **A10**–**A12**). The green bars represent the experimental binding free energies (Δ*G*_exp_^o^) of the test set compounds, while gray, yellow, and red bars depict their average binding free energy values over four MD trajectory samplings (100–200, 250–350, 400–500, and 20–500 ns) as retrieved by the various LIE methods utilized. The bars with intense tones correspond to calculation methods that do not consider the SASA parameter, while those represented in pale tones correspond to the methods that include the SASA parameter. The error bars correspond to the standard deviation (SD) values calculated over four trajectory samplings.

For this purpose, the non-bonded van der Waals and electrostatic interaction energy terms were calculated from the resulting MD production trajectories (sampled at four time ranges: 100–200, 250–350, 400–500, and 20–500 ns) by employing three different calculation methods: *cpptraj* lie command,^38^ LIEW,^39^ and VMD’s NAMD Energy plugin,^40^ with and without considering SASA (Supporting Information, Table S3). Based on the average calculated binding affinity with the lowest SD value (Δ*G*_pred_avg_) of the test set consisting of four ligands (Figure 10; **A06**, **A10**–**A12**), the “NAMD_no sasa_” method was selected as the most appropriate one (Supporting Information, Tables S3 and S4), which was subsequently used to calculate the relative free energies of binding (Δ*G*_bind_pred_^°^) of the external NBTIs (**L01**–**L10**) with correctly predicted binding affinities by the quasi-atomistic surrogate models (Table 2). It should be highlighted that the LIE weighting parameters corresponding to the selected “NAMD_no sasa_” calculation method (α = 0.16, β = 0.029, γ = 0.0, intercept = −1.72, and RMSE = 1.17) were derived solely on NBTI ligands active against the *S. aureus* DNA gyrase enzyme. However, considering the high level of conservancy of amino acid residues outlining the NBTI binding pocket in both enzymes (*S. aureus* and *E. coli* DNA gyrase), we assumed that the same “NAMD_no sasa_” method and its corresponding LIE fitting parameters might be applicable for prediction of binding free energies of NBTIs against the *E. coli* DNA gyrase enzyme, as well.

Table 3 summarizes the experimental and predicted binding free energy values of selected NBTI compounds (**L01**–**L10**) for both enzymes (*S. aureus* and *E. coli* DNA gyrase) as derived by utilizing the “NAMD_no sasa_” method. For most of the ligands, the accuracy of predicted binding free energy values (Δ*G*_bind_pred_^°^) deviates around ±1.0 kcal/mol from their experimental values (Δ*G*_bind_exp_) against *S. aureus* DNA gyrase (Table 4). Nonetheless, it should be emphasized that although predictions of the binding free energies of NBTIs against the *E. coli* DNA gyrase enzyme are not so satisfactory, they are accurate enough considering the matter that the calibration of LIE weighting parameters was grounded solely on *S. aureus* DNA gyrase-NBTI complex systems.

**Table 3:** Experimental (Δ*G*_bind_exp_) and Predicted (Δ*G*_bind_pred_^°^) Binding Free Energy Values of Selected External Compounds (Table 2, **L01**–**L10**) against Both Enzymes (*S. aureus* and *E. coli* DNA Gyrase, Respectively) as Derived by the Selected LIE Method (“NAMD_no sasa_”; α = 0.16, β = 0.029, γ = 0.0, Intercept = −1.72)

*S. aureus* DNA gyrase			*E. coli* DNA gyrase		
ID	Δ*G*_bind_exp_ (kcal/mol)	Δ*G*_bind_pred_^°^ (kcal/mol)	ID	Δ*G*_bind_exp_ (kcal/mol)	Δ*G*_bind_pred_^°^ (kcal/mol)
**L01**	–9.08	–9.19	**L05**	–8.94	–10.26
**L02**	–8.01	–9.36	**L06**	–9.19	–10.48
**L03**	–9.06	–9.82	**L08**	–8.90	–9.74
**L04**	–9.96	–9.65	**L09**	–7.75	–10.22
**L05**	–11.15	–9.53	**L10**	–8.62	–10.68
**L06**	–9.96	–9.22			
**L07**	–9.77	–9.61			
**L08**	–10.35	–9.49			

**Table 4:** Comparison of ΔΔ*G*_bind_^°^ for Both Methods (Multidimensional QSAR, mQSAR and LIE) for *S. aureus* and *E. coli* DNA Gyrase^a^

	ΔΔ*G*_bind_^°^ (kcal/mol)			
	*m*QSAR		LIE method	
ID	*S. aureus*	*E. coli*	*S. aureus*	*E. coli*
**L01**	0.11	0.45	0.11	
**L02**	0.00	0.74	1.35	
**L03**	0.14	1.24	0.76	
**L04**	0.17	0.86	0.31	
**L05**	0.86	0.27	1.62	1.32
**L06**	0.44	0.07	0.74	1.29
**L07**	0.28	0.36	0.16	
**L08**	0.31	0.11	0.86	0.84
**L09**	1.08	0.07		2.47
**L10**	0.45	0.19		2.06

a ΔΔ*G*_bind_^°^ = |Δ*G*_bind_exp_^°^ – Δ*G*_bind_pred_^°^|.

As demonstrated in Table 4, the maximum deviation in the prediction of binding free energies can be attributed to the compound **L05** for *S. aureus* DNA gyrase (1.62 kcal/mol), i.e., **L09** for *E. coli* DNA gyrase (2.47 kcal/mol). However, it is evident that both methods (the multidimensional quasi-atomistic QSAR and LIE) provide correct predictions of the binding free energies (Δ*G*_bind_pred_^°^) of **L01**–**L10** NBTIs, which are sufficiently accurate relative to their experimental values (Δ*G*_bind_exp_) considering their structural diversity and can effectively be applied for prediction of binding affinities of *de novo* designed/optimized NBTIs against the DNA gyrase enzyme originating from Gram-positive (e.g., *S. aureus*) and Gram-negative (e.g., *E. coli*) bacterial species.

## 3 Conclusions

In this article, we present the construction and validation of comprehensive multidimensional predictive NBTI’s binding site surrogate models of DNA gyrase enzymes originating from *S. aureus* and *E. coli* organisms. Both multidimensional models (*q*^2^ = 0.791 for *S. aureus* DNA gyrase and *q*^2^ = 0.806 for *E. coli* DNA gyrase) exhibit good predictive performance as demonstrated by the accuracy in predicting the binding affinities of an independent external set of 26 NBTIs (compiled from our recent publications) not used for construction of the models (*p*^2^ = 0.761 for *S. aureus* DNA gyrase and *p*^2^ = 0.677 for *E. coli* DNA gyrase; *p*^2^, predictive *r*^2^). The sensitivity of the models to the biological data used (IC_50_) was verified by conducting 20 scramble tests. Moreover, the evaluation of quasi-atomistic properties populating the binding site surrogates of both DNA gyrase enzymes correctly mirrored some of the key amino acid residues (e.g., Ala68, Met75, Asp83, and Met121 in *S. aureus* DNA gyrase, i.e., Ala67, Met74, Asp82, and Met120 in *E. coli* DNA gyrase), which are of cardinal importance for the NBTI’s binding and affinity.

Furthermore, a subset of the external NBTIs (**L01**–**L10**) with highly predicted binding affinities against both DNA gyrase enzymes (e.g., **L01**–**L08** for *S. aureus* DNA gyrase, i.e., **L05**, **L06**, and **L08**–**L10** for *E. coli* DNA gyrase) was subjected to extensive MD simulations for investigating their binding and dynamic profile. Hydrogen-bonding occupancy analysis of the studied NBTIs revealed that the majority of them establish a direct ionic interaction with Asp83, i.e., Asp82 residue from a sole GyrA subunit as well as a water-mediated ionic interaction with Asp83, i.e., Asp82 residue from the second GyrA subunit in both organisms over the entire simulation time; this finding is firmly corroborated with the experimental crystallographic evidence.^13^

In contrast to Asp83/Asp82 residues crucial for NBTI potency that are solvent-exposed as confirmed by the calculated SASA values (Supporting Information, Figure S2), the amino acid residues delineating the NBTI’s binding pocket (e.g., Ala68, Gly72, Met75, and Met121 in *S. aureus* DNA gyrase, i.e, Ala67, Gly71, Met74, and Met120 in *E. coli* DNA gyrase) are mainly hydrophobic in nature, i.e., properties which are well emulated by the pseudoatomistic multidimensional DNA gyrase models. These amino acid residues establish a network of hydrophobic contacts with the NBTI’s RHS fragments and additionally contribute to the low nanomolar enzyme inhibitory potency of this class of antibacterials as portrayed by their *in vitro* enzyme inhibitory potencies (Table 2).^26,32^

In addition, the binding affinities of selected, external NBTI ligands (**L01**–**L10**) were quantified by utilizing the LIE method directly from their MD production trajectories, as well. Since the default LIE weighting parameters (α = 0.5, β = 0.16, and γ = 0.0) are apparently not applicable for calculating binding free energies (Δ*G*_bind_pred_^°^) of our systems, we were forced to derive a new set of fitting parameters (α = 0.16, β = 0.029, γ = 0.0). Considering the high level of conservancy of amino acid residues delineating the NBTI’s binding pocket in *S. aureus* and *E. coli* DNA gyrase, only the data acquired from MD simulations conducted on *S. aureus* DNA gyrase complexes were used for deriving the fitting parameters, which were efficiently applied for calculating the NBTI’s Δ*G*_bind_pred_^°^ in the *E. coli* DNA gyrase enzyme. Both methods, the multidimensional quasi-atomistic surrogate models and LIE, provide relatively accurate predictions of the binding free energies (Δ*G*_bind_pred_^°^) of the selected NBTI analogues relative to their experimental values (Δ*G*_bind_exp_) that differ around ±1.0 kcal/mol. Accordingly, these two modeling strategies can effectively be used for predicting the binding affinities of any *de novo* designed/optimized NBTI against the DNA gyrase enzyme originating from Gram-positive (e.g., *S. aureus*) and Gram-negative (e.g., *E. coli*) bacterial pathogens. We are convinced that the results conferred in this study would be beneficial in the current NBTI’s hit-to-lead pipelines for design and optimization of NBTI antibacterials that are effective in combating bacterial resistance.

## 4 Materials and Methods

### 4.1 NBTI Chemical Libraries

Considering the well-established structure–activity relationships (SAR) guidelines of currently known NBTI antibacterials (Figure 1a), two chemical libraries comprising 199 and 133 structurally diverse NBTIs, with experimentally determined *in vitro* antibacterial potencies against *S. aureus* DNA gyrase (IC_50_ = 0.007–50 μM) and *E. coli* DNA gyrase (IC_50_ = 0.020–100 μM), respectively (hereafter named as NBTI_SA_ and NBTI_EC_), were compiled from the literature.^10,25,28,41−49^ The chemical structures comprising both NBTI libraries were initially sketched by using the ChemDraw Professional 20.1.1 suite and subsequently energetically minimized utilizing Discovery Studio’s integrated Merck molecular force field (MMFF) module.^50,51^

### 4.2 Molecular Docking Calculations

To account for induced fit of investigated NBTI antibacterials within *S. aureus* and *E. coli* DNA gyrase NBTI’s intercalating site, the NBTI libraries (NBTI_SA_ and NBTI_EC_) were subjected to flexible molecular docking calculation campaigns using the GOLD docking suite.^52^ For this purpose, our recently disclosed crystal structure of *S. aureus* DNA gyrase–DNA-AMK12 complex (PDB ID: 6Z1A)^12^ and cryo-EM structure of *E. coli* DNA gyrase–DNA-gepotidacin complex (PDB ID: 6RKS)^18^ were employed. The experimental coordinates of the AMK12 ligand, i.e., gepotidacin in *S. aureus* and *E. coli* DNA gyrase, were used for defining the NBTI’s binding site (cavity radius of 15.5 Å) in both enzymes. The amino acid residues Met75, Asp83, and Met121 in *S. aureus* GyrA and Ile74, Asp82, and Met120 in *E. coli* GyrA were considered flexible during the docking calculations (Figure 1b). The molecular docking protocol was initially validated, by conducting threefold redocking validation trials of the natively present AMK12, i.e., gepotidacin conformation within its corresponding DNA gyrase binding site in *S. aureus*, i.e., *E. coli*. As a key determinant for the well-conducted ligand’s reproduction as well as the goodness of all performed structure-based settings, the heavy-atom root-mean-square deviation values (RMSD ≤ 2.0 Å) between redocking-derived solutions and AMK12, i.e., gepotidacin ligand conformation, were calculated (see the Supporting Information, Table S5 and Figure S4).^52^ In order to sample a much wider conformational space of investigated NBTIs, each compound comprising NBTI_SA_ and NBTI_EC_ library, respectively, was flexibly docked up to 100 times within the corresponding binding site of *S. aureus*, i.e., *E. coli* DNA gyrase, by employing the same settings and parameters of the GOLD genetic algorithm (population size = 100, selection pressure = 1.1, number of operations = 100,000, number of islands = 5, niche size = 2, migrate = 10, mutate = 95, crossover = 95). The quality of the docking-derived NBTI’s binding poses was quantified utilizing the GoldScore Fitness function.^53^

### 4.3 Construction of the 4D Ligand’s Representation

The multidimensional binding-site surrogate modeling in a broader sense can be regarded as an evolved three-dimensional (3D) QSAR concept considering the widely accepted ligand-target induced-fit paradigm.^54,55^ This requires a proficient implementation of a so-called 4D formalism in the conformational sampling/representation of the binding species (the ligands).^56^ The 4D representation of ligand molecules is substantiated on the idea that each ligand might be expressed as a 3D spatial ensemble of various conformers, orientomers, or protomeric forms, which in turn significantly contributes in minimizing the bias connected with the ligand’s binding hypothesis. Consequently, the needed 4D representation of experimental NBTIs comprising the NBTI_SA_ and NBTI_EC_ library, respectively, was achieved in three consecutive steps:

(i) Identification of the minimum-energy NBTI conformation from each cluster of 100 docked NBTI poses corresponding to each NBTI ligand separately.
(ii) Assembling of ligand ensembles comprising usually 5–8 NBTI docked poses from each cluster selected within an energy framework of ±5.0 kcal/mol around the previously identified minimum-energy NBTI conformations.
(iii) Flexible alignment of thus assembled NBTI conformers over the bioactive co-crystallized AMK12, i.e., cryo-EM gepotidacin conformation (the templates).

The input parameters critical for multidimensional induced-fit binding-site simulations were calculated on the prepared 4D ligand’s data representing NBTI_SA_ and NBTI_EC_ libraries, in a concomitant fashion by constructing an efficient one-step Pipeline Pilot protocol.^57^ AMSOL 7.1 was utilized for calculation of the NBTI’s CM1 partial atomic charges and SM2 solvation energies,^56^ while their internal strain energies were computed using DMol^3^ engine.^58^ Moreover, the experimental inhibitory potencies of NBTI_SA_ and NBTI_EC_ antibacterials originally expressed as IC_50_ (μM) were converted to molar concentrations (M) for deriving and fitting their free energies of binding during the simulations. The obtained data served as an input for multidimensional QSAR modeling.

### 4.4 Multidimensional QSAR Modeling

The NBTI’s quasi-atomistic binding-site surrogate modeling of *S. aureus* and *E. coli* DNA gyrase enzyme, respectively, was conducted by using the Quasar^X^ 6.2 platform that allows construction and validation of genetic algorithm-optimized 6D-QSAR models.^59,60^ In Quasar^X^, the ligand’s binding site is represented as a surrogate, which is a 3D quasi-atomistic pseudosurface covering a series of superimposed, bioactive conformations of the binding entities (4D ligand’s representation) at van der Waals distance.^61^ The topology of such a pseudosurface emulates the 3D spatial shape of the ligand’s binding site that is capable to simulate a local induced fit in an aqueous environment through dynamic adaptation to the ligand’s conformations. In Quasar^X^, this is allowed by scaling the dimensionality of the initial 4D ligand’s representation through concomitant consideration of various induced-fit scenarios (5D-QSAR)^61−64^ as well as introduction of diverse solvation models (6D-QSAR).^59^ Moreover, the pseudosurface is occupied by a variety of color-coded quasi-atomistic properties (e.g., hydrogen-bonding donors and acceptors, flip-flop hydrogen-bonding entities, salt bridges, and hydrophobic properties) that correspond to different structural functionalities of amino acid residues delineating the binding site at the true biological target, which in turn enable a qualitative evaluation of the crucial ligand–protein interactions. The modeling procedure is grounded on generating a family of 200 or more quasi-atomistic binding-site surrogates (parent models derived from the training set) averaged during the simulation using a genetic algorithm. The relative free energy of ligand’s binding (*E*_bdg_) is estimated for the average binding site surrogate model as
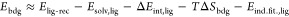
where *E*_lig-rec_ denotes the enthalpic contribution to the ligand–protein interaction determined by the Quasar^X^ directional force field,^65^*E*_solv,lig_ is the ligand desolvation energy, Δ*E*_int,lig_ and *T*Δ*S*_bdg_ refer to the change in the ligand’s internal energy and entropy, respectively, upon its binding to the protein target, and *E*_ind.fit.,lig_ is the energy uptake required for dynamic adaptation of the average pseudosurface. Since Quasar^X^ deals with the 4D ligand’s data, the energy contribution of each individual ligand conformation (*E*_bdg,ind_) to the total energy (*E*_bdg,tot_) is weighted by a normalized Boltzmann factor (*w*_*i*_)^59,66^
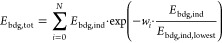

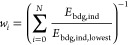
The binding free energies of the ligands (Δ*G*_pred_^o^) are then calculated by means of a cross-validated linear regression between their experimental free energy (Δ*G*_exp_^o^) and the estimated relative free energy (*E*_bdg_) considering solely the ligands comprising the training set
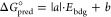
where fitting coefficients *a* and *b* denote the slope and intercept, respectively, which are an integral part of a specific binding site surrogate model and can subsequently be applied for estimation of the relative binding free energy of new ligands not used for the development of the model.

Following the classical QSAR modeling protocols, the NBTI libraries (NBTI_SA_ and NBTI_EC_) containing 4D ligand data for *S. aureus* and *E. coli* DNA gyrase enzyme, respectively, were divided into training and test sets (in percentage ratios of 80 and 20%, respectively) prior to the modeling procedure by taking into account their maximum scaffold dissimilarity and binding affinity. Two comprehensive quasi-atomistic NBTI binding site surrogate models of *S. aureus* and *E. coli* DNA gyrase enzyme were constructed separately in an evolutionary fashion starting from an initial family of 200 parent models by using the Quasar^X^ partial least-squares genetic algorithm (PLS-GA) modeling methodology.^67^ The flexibility of both enzymes was emulated by considering six induced-fit scenarios, as implemented in Quasar^X^. During the modeling, the models were internally validated by weighted cross-validation leave-n-out (*n* = 3) procedure (*q*^2^)^54,68^ as well as externally by estimation of the predictive squared correlation coefficient for the test set molecules (*p*^2^; predictive *r*^2^ for the test set). To account for transcription of mapped quasi-atomistic properties on the pseudosurface during the modeling, a fixed mutation rate of 0.02 was used. The sensitivity of established and validated *S. aureus* and *E. coli* DNA gyrase NBTI binding site surrogate models toward the biological data used (IC_50_) was evaluated by performing 20 scramble (*Y*-randomization) trials.^69^ It should be stressed that in both cases, the training set molecules were used solely for constructing the *S. aureus* and *E. coli* DNA gyrase surrogate models, while their predictive power was estimated by employing the test set molecules that were not used for the development of the models. Lastly, the validity of both models was additionally challenged by predicting the binding affinities for an external set of 26 NBTI antibacterials with *in vitro* experimentally determined inhibitory potencies for *S. aureus* and *E. coli* DNA gyrase from our previous studies.^26,32,35,36^

### 4.5 Molecular Dynamics Simulations

All-atom molecular dynamics (MD) simulations on the investigated DNA gyrase-DNA-NBTI complex systems were performed using the AMBER20 package.^70^ The AMBER-ff14SB^71^ and DNA-OL15^72^ force fields were used to parametrize the proteins and DNA, respectively. Partial atomic charges of investigated, geometry-optimized NBTI ligands were calculated with the Gaussian16 package^73^ by performing Merz–Kollman’s population analysis at the Hartree–Fock level of theory using the 6-31G* basis set, while for those NBTIs containing iodine, a mixed 6-31G*/3-21G basis set was used. To derive the restrained electrostatic potential (RESP) charges as well as the other ligand’s force field parameters, the bond lengths and angles obtained from the NBTI optimized geometries were employed by utilizing the Antechamber module of AMBER20.^74^ The parametrized systems were initially neutralized by addition of Na^+^/Cl^–^ counterions^75^ and then immersed in a 10 Å cubic box (128 Å × 130 Å × 112 Å) of TIP3P explicit water molecules,^76^ which resulted in ∼186,461 atoms per simulation system. Prior to running MD simulations, all assembled systems were first minimized by using steepest descent energy minimization to circumvent any existing van der Waals clashes between the atoms as well as to fix the poor geometries of protein side chain residues. The integrity of the simulation systems was assured by conducting an extensive equilibration on the fully restrained systems via two-step heating from 0 to 150 K for 2 ns and from 150 to 303 K for the next 2 ns, followed by an additional 10 ns of unrestrained *NPT* equilibration. 500 ns MD production simulations were performed with the canonical (*NVT*) ensemble using periodic boundary conditions on fully unrestrained systems at a time step of 2 fs. The particle-mesh Ewald method^77^ was used to account for the long-range electrostatic interactions. The analysis of the obtained MD production trajectories [calculation of RMSD and radius of gyration (*R*_g_), the NBTI-gyrase hydrogen-bonding analysis, contact analysis, and the analysis of solvent-accessible surface area (SASA)] for each simulated system (deprived of water and counterions) was conducted by utilizing the VMD software package^78^ and *cpptraj* module of Ambertools 20.^70^

### 4.6 Linear Interaction Energy Calculations

The estimation of relative free energy of the ligand’s binding (Δ*G*_bind_^°^) is of paramount importance in the rational design of new drug molecules.^79^ Nowadays, there is a plethora of highly accurate, force field-grounded free energy calculation methods directly from MD simulations, of which the alchemical ones such as free energy perturbation (FEP)^80^ and thermodynamic integration (TI)^81^ are frequently utilized. Indeed, these methodologies proved highly rigorous in estimating Δ*G*_bind_^°^; however, they were found computationally more expensive in sampling a variety of non-physical, intermediate states (e.g., protein, ligand, and solvent configurational spaces) and consequently less practical for fast computation of the relative ligand’s binding free energies.^82^ In contrast, the end-point approaches such as the linear interaction energy (LIE) method allow relatively fast and accurate calculation of Δ*G*_bind_^°^ by explicit sampling of the ligand, protein–ligand, and solvent configurational space considering two states only, i.e., protein–ligand bound and unbound states.^37^ In the LIE approach, which is substantiated on the linear response approximation (LRA) theory,^83,84^ Δ*G*_bind_^°^ of a ligand is presumed to be in a linear correlation to differences in the van der Waals and electrostatic interactions (Δ*V*_vdW_ and Δ*V*_Ele_, respectively) between the ligand and its surrounding derived from MD simulations of ligand–protein bound and unbound states in the explicit solvent. The differences in non-bonded ligand interaction energy terms (Δ*V*_vdW_ and Δ*V*_Ele_) are scaled by the fitting parameters α and β, respectively^85,86^
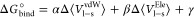

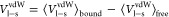

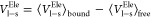
where ⟨*V*_l–s_^vdW^⟩ and ⟨*V*_l–s_^Ele^⟩ are MD-derived averages of the non-bonded van der Waals (vdW) and electrostatic (Ele) interactions of a ligand with its surrounding, respectively, Δ denotes the change in these averages obtained by simulating the ligand in its free state (simulation of the ligand in solution only) and bound state (simulation of the ligand bound to its solvated target), and γ is an offset parameter usually related to the surface area.^54^ While default values for the fitting parameters α = 0.5 and β = 0.16 (and optionally, γ = 0) frequently give reasonable Δ*G*_bind_^°^ predictions for a variety of systems,^85,87−89^ it was determined that they are not universally applicable for any system.^90^ Put differently, the α and β fitting parameters are proposed to be freely adjustable and can be obtained by fitting to experimentally determined Δ*G*_bind_^°^ values for a specific set of ligand–protein systems.^91,92^

Considering the high level of structural resemblance, in particular, the conservancy of amino acid residues delineating the NBTI’s binding site in Gram-positive (e.g., Ala68, Met75, Asp83, and Met121 in *S. aureus* GyrA) and Gram-negative (e.g., Ala67, Ile74, Asp82, and Met120 in *E. coli* GyrA) bacteria,^14^ LIE parameters (α, β, and γ) were derived solely from MD simulations conducted on *S. aureus* DNA gyrase–NBTI systems, assuming that the same would be applicable for Δ*G*_bind_^°^ predictions of NBTIs targeting *E. coli* DNA gyrase, as well. For this purpose, 18 structurally diverse NBTIs (IC_50_ = 0.007–4.7 μM)^12,28,31,41,42,45,46,49^ from the NBTI_SA_ library were selected (see the Supporting Information, Table S2) and subjected to 500 ns MD simulations. For each NBTI ligand, two concomitant MD simulations were performed (free NBTI and DNA gyrase–NBTI complex in an aqueous environment), whereas our recently disclosed crystal structure of *S. aureus* DNA gyrase (PDB ID: 6Z1A)^12^ was employed for assembling the initial DNA gyrase–NBTI simulation systems. The calibration of LIE parameters was done on the experimental Δ*G*_bind_exp_ values for the training systems utilizing the ordinary least-squares (OLS) fitting procedure as implemented in the Python *scikit*-learn 0.17 package^67,68^ and validated by predicting Δ*G*_bind_pred_^°^ values of the test systems. To minimize the uncertainty in fitting of the LIE parameters and consequently predicted Δ*G*_bind_pred_^°^ values, the non-bonded van der Waals and electrostatic interaction energy terms were calculated from the resulting MD production trajectories sampled at four different time ranges (e.g., 100–200, 250–350, 400–500, and 20–500 ns) by employing three different calculation methods: Ambertools *cpptraj* lie command,^38^ Ambertools linear interaction energy workflow (LIEW) module,^39^ and VMD’s NAMD Energy plugin.^40^ Standard deviation (SD) of the predicted relative binding free energies averaged over all four trajectory samplings (Δ*G*_pred_avg_) was used as a decisive criterion in selection of the most appropriate LIE method, which was subsequently used to reproduce the relative free energies of binding (Δ*G*_bind_pred_^°^) of those external NBTIs with correctly predicted binding affinities by the quasi-atomistic surrogate models.

## Data Availability

The initial chemical libraries of NBTI antibacterials (e.g., NBTI_SA_ and NBTI_EC_ in the *.sdf format) as well as 4D ligand ensembles of both libraries together with their experimentally determined *in vitro* enzyme inhibitory potencies ready to be used for multidimensional QSAR modeling used in this study are freely available online at https://zenodo.org/records/10440738.

## Supporting Information Available

Evolution detail of *S. aureus* and *E. coli* DNA gyrase multidimensional QSAR models, scrambling trials for evaluation of sensitivity of multidimensional QSAR models, total solvent-accessible surface area (SASA) plots and average SASA per residue plots, dataset of 18 structurally diverse NBTIs and analysis of MD production trajectories for fitting of the LIE weighting parameters (α, β, and γ), detailed statistical data for all six LIE methods employed (“*cpptraj*_no sasa_/_sasa_”, “NAMD_no sasa_/_sasa_”, and “LIEW_no sasa_/_sasa_”) calculated from the obtained MD production trajectories sampled at four different time ranges (e.g., 100–200, 250–350, 400–500, and 20–500 ns) for the training and test sets, correlation of experimental (Δ*G*_bind_exp_) and predicted (Δ*G*_bind_pred_^°^) relative binding free energy values derived utilizing the selected “NAMD_no sasa_” LIE method from the resulting MD production trajectories sampled at a time range of 20–500 ns, and molecular re-docking validation data (PDF)
