# Supplementary material for: Dynamic Profiling and Binding Affinity Prediction of NBTI Antibacterials against DNA Gyrase Enzyme by Multidimensional Machine Learning and Molecular Dynamics Simulations
Source: ACS Omega. 2024 Apr 11;9(16):18278–95. doi: 10.1021/acsomega.4c00036 (PMC11044241; doi:10.1021/acsomega.4c00036)
Supplement: Supplementary file 1 — ao4c00036_si_001.pdf [file ao4c00036_si_001.pdf]

Supporting Information

Dynamic profiling and binding affinity prediction of NBTI antibacterials against DNA gyrase enzyme by multidimensional machine learning and molecular dynamics simulations

Maja Kokot [1,2], and Nikola Minovski[1,*]

[1] Theory Department, Laboratory for Cheminformatics, National Institute of Chemistry, Hajdrihova 19, 1001 Ljubljana, Slovenia

[2] Department of Pharmaceutical Chemistry, Faculty of Pharmacy, University of Ljubljana, Aškerčeva cesta 7, 1000 Ljubljana, Slovenia

[*]Corresponding author: Tel. +386 1 4760 383, e-mail: nikola.minovski@ki.si

Evolution of *S. aureus* and *E. coli* DNA gyrase multidimensional QSAR models

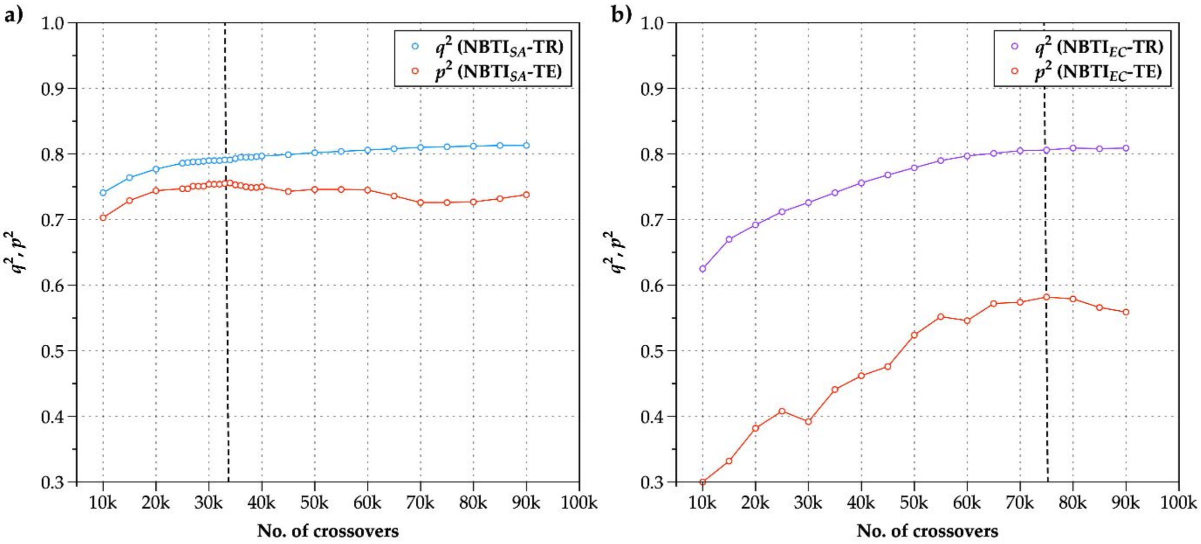

Figure S1. a) Evolution of the model family for *S. aureus* DNA gyrase: q^2 (blue dots) are cross-validated r^2 values for the training set (q^2=0.791) and p^2 (red dots) represent predictive r^2 values for the test set (p^2=0.756) obtained at 34000 crossovers. **b)** Evolution of the model family for *E. coli* DNA gyrase: q^2 (violet dots) are cross-validated r^2 values for the training set (q^2=0.806), while p^2 (red dots) denotes r^2 values for the test set (p^2=0.582) reached at 75000 crossovers.

Scrambling (*Y*-randomization) trials for evaluation of sensitivity of multidimensional QSAR models to the biological data employed.

Table S1. Details of the 20 scramble tests

**Scramble trial**	***S. aureus* DNA gyrase**			***E. coli* DNA gyrase**		
	q^2	r^2	p^2	q^2	r^2	p^2
**1**	0.248	0.263	-0.322	0.416	0.424	-0.544
**2**	0.362	0.366	-0.068	0.390	0.422	-0.194
**3**	0.228	0.235	-0.543	0.383	0.397	-0.684
**4**	0.269	0.277	-0.099	0.454	0.467	-0.900
**5**	0.261	0.278	-0.580	0.379	0.385	-0.683
**6**	0.400	0.410	-0.478	0.473	0.488	-0.827
**7**	0.247	0.256	-0.026	0.033	0.000	-0.097
**8**	0.341	0.357	-0.206	0.469	0.478	-0.528
**9**	0.231	0.249	0.038	0.346	0.352	-0.520
**10**	0.248	0.263	-0.032	0.113	0.427	-0.563
**11**	0.225	0.244	0.027	0.372	0.381	-1.379
**12**	0.309	0.312	-0.037	0.298	0.324	0.397
**13**	0.372	0.374	0.108	0.025	0.000	-0.095
**14**	0.355	0.360	-0.604	0.310	0.331	0.058
**15**	0.002	0.000	-0.034	0.279	0.295	0.061
**16**	0.305	0.314	-0.203	0.420	0.425	-0.741
**17**	0.234	0.237	-0.142	0.293	0.309	-0.639
**18**	0.303	0.313	-0.296	0.460	0.474	-0.457
**19**	0.004	0.000	-0.034	0.511	0.521	-0.431
**20**	0.245	0.256	-0.238	0.424	0.434	-0.501
**Average**	**0.259**	**0.268**	**-0.188**	**0.342**	**0.367**	**-0.463**

r^2: model's correlation coefficient, q^2: model's cross-validated r^2, p^2: predictive r^2 for the test set.

Total Solvent-accessible surface area (SASA) plots and average SASA *per* residue plots of 500 ns MD-simulated NBTI compounds (L01-L10).

The total solvent-accessible surface area (SASA) plots of the *apo* (ligand-free form; Gyr$_{apo}$) form and NBTI-ligated *S. aureus* DNA gyrase (e.g., Gyr-**L01-L08**) and *E. coli* DNA gyrase (e.g., Gyr-**L05**, Gyr-**L06**, and Gyr-**L08-L10**) systems, respectively, show that the bound NBTI ligands stabilize the complexes (Figure S2, upper plots). It is also notable that SASA of the *apo* (ligand-free) forms of both enzymes is significantly lower compared to NBTI-ligated systems, suggesting that the binding of the ligands increase the area of the protein that is accessible to water. In both enzymes, Asp83, i.e., Asp82 and Met121, i.e., Met120 contribute the most to the total SASA, indicating that amino acid residues Ala68, Gly72, and Met75 in *S. aureus* DNA gyrase, i.e., Ala67, Gly71, and Ile74 in *E. coli* DNA gyrase delineate a tight hydrophobic binding pocket that cannot be exposed to any water molecule (Figure S2, bottom plots).

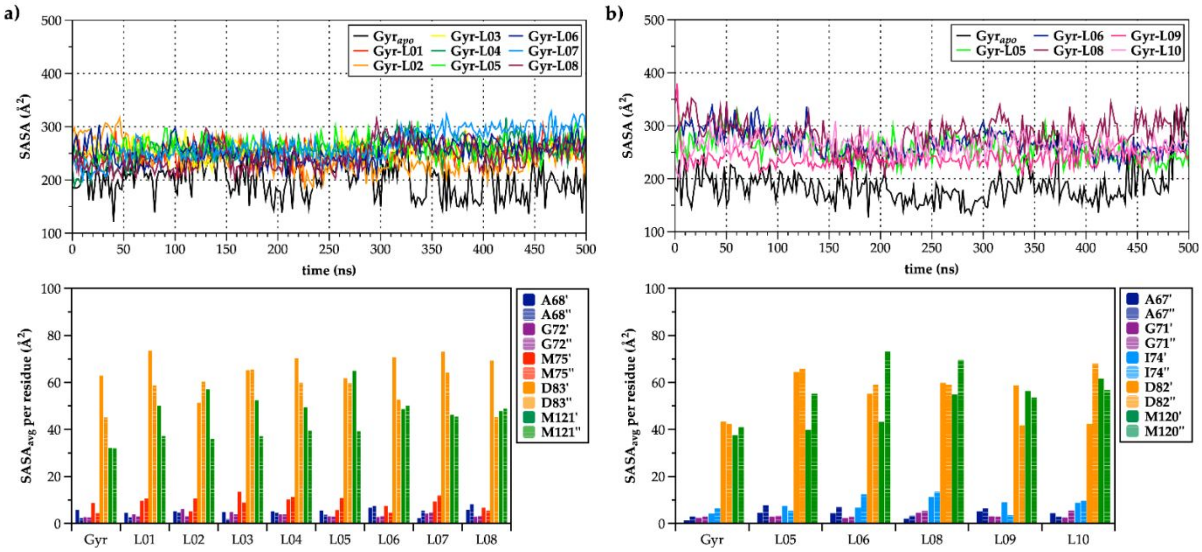

Figure S2. Solvent-accessible surface area (SASA [Å^2]) plots of 500 ns MD-simulated *apo* (ligand-free) *S. aureus* and *E. coli* DNA gyrase enzymes and their NBTI-ligated complexes (Gyr-**L01-L08, i.e.,** Gyr-**L05**, Gyr-**L06**, and Gyr-**L08-L10**). Upper plots: total SASA as a function of the simulation time, and Bottom plots: average SASA *per* residue for: **a)** *S. aureus* DNA gyrase, and **b)** *E. coli* DNA gyrase. The solid bars depict the average SASA values over the entire simulation time of amino acid residues from one GyrA subunit, while the striped bars are related to the average SASA values over the entire simulation time of amino acid residues from the other GyrA subunit. All SASA values (total and *pre* residue) are calculated utilizing the Shrake–Rupley algorithm [1].

A dataset of 18 structurally diverse NBTIs selected from the NBTI$_{SA}$ library for fitting of the LIE parameters (α, β, and γ).

Table S2. Chemical structures, experimental bioactivity data, and division of 18 NBTIs (training/test set) selected from the NBTI$_{SA}$ library for derivation of LIE fitting parameters.

ID	Structure	*S. aureus* DNA gyrase	
		**$IC_{50,exp}$ (μM)**	**$\Delta G^{\circ}_{bind\_exp}$ (kcal/mol)**
**A01**†	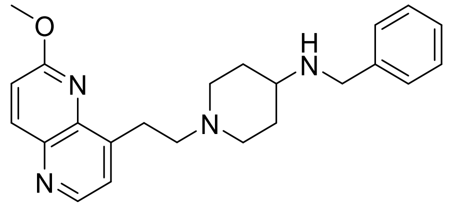	1.020	-8.306
**A02**†	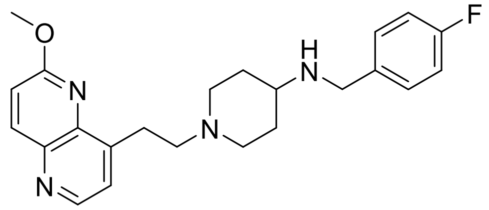	0.550	-8.678
**A03**†	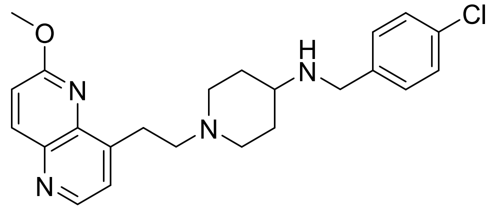	0.035	-10.34
**A04**†	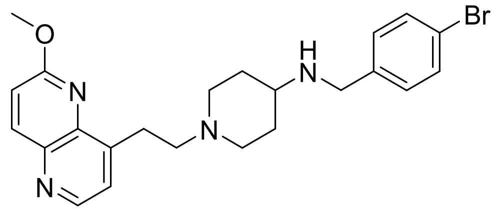	0.011	-11.31
**A05**†	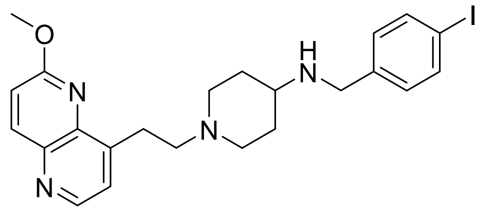	0.007	-11.03
**A06**‡	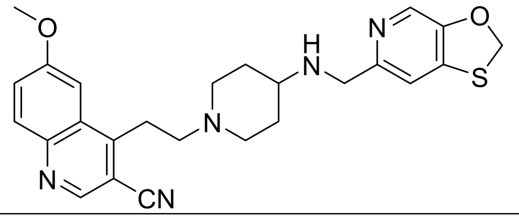	0.014	-10.89

**A07**[†]	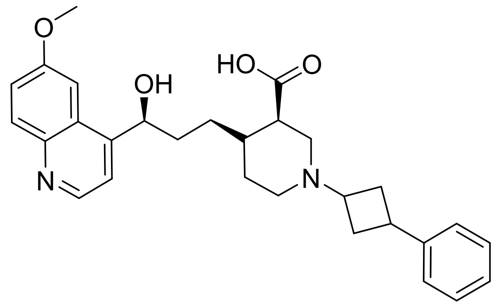	4.7	-7.386
**A08**[†]	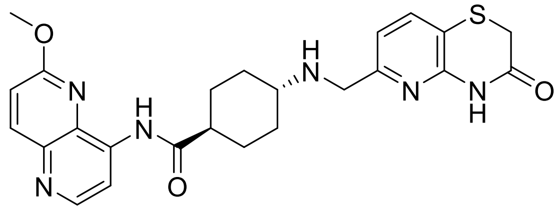	0.008	-11.23
**A09**[†]	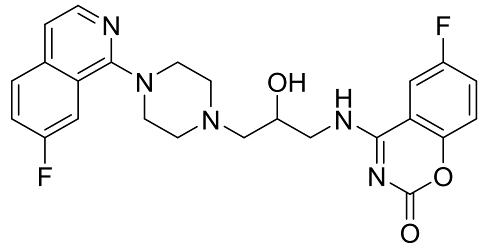	0.02	-10.67
**A10**[‡]	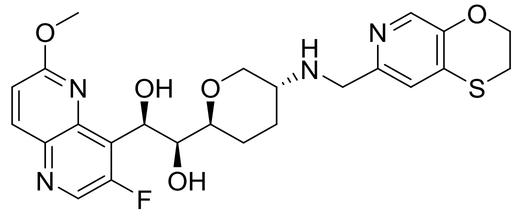	0.03	-10.43
**A11**[‡]	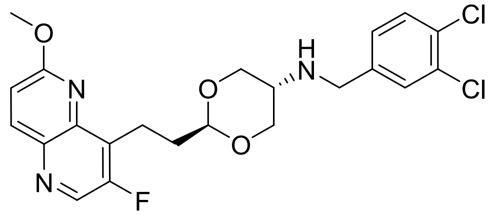	0.04	-10.26
**A12**[‡]	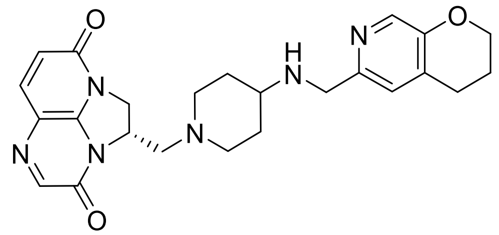	0.047	-10.16
**A13**[†]	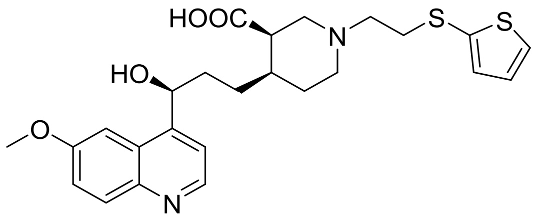	2.42	-7.786

**A14**†	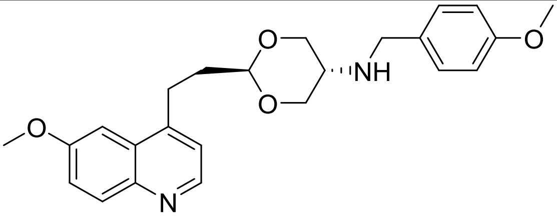	1.5	-8.074
**A15**†	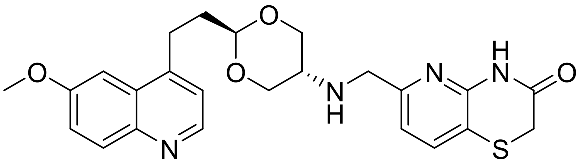	0.59	-8.636
**A16**†	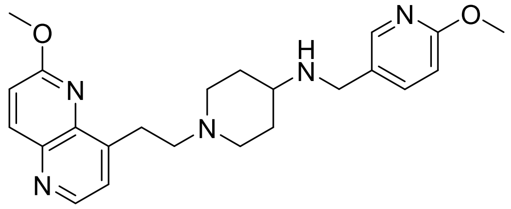	0.34	-8.968
**A17**†	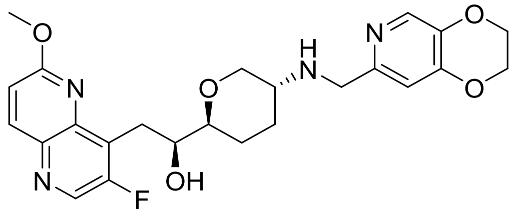	0.125	-9.570
**A18**†	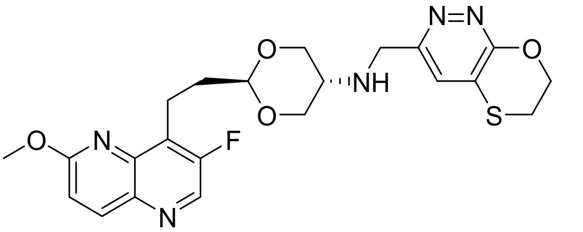	0.07	-9.919

† training set ligands (n=14), ‡ test set ligands (n=4).

Analysis of MD simulation trajectories for 18 selected systems (*S. aureus* DNA gyrase-DNA-NBTI$_{SA}$ complexes; A01-A18) utilized for derivation of the LIE fitting parameters (α, β, and γ).

The resulting MD trajectories of 18 selected DNA gyrase-DNA-NBTI$_{SA}$ systems were initially analyzed by monitoring their root-mean-square deviations (RMSD) and radius of gyration (Rg) that account for their stability and compactness (Figure S3). The RMSD values were calculated for each entity of the complex systems separately (e.g., protein backbone, DNA, and NBTI ligands) as well as for the system in the absence of NBTI ligands (Gyr$_{apo}$, i.e., DNA$_{apo}$ form). As depicted on Figure S3a, the stability of all the complexes is established by no significant RMSD deviations (~1.5-3.0 Å). When compering the RMSD values of DNA (Figure S3b), one can easily percieve a higher DNA stability for the complex systems (~1.0-2.0 Å) relative to the ligand-free system (~1.0-3.0 Å; *apo* form) indicating to stabilization of the DNA upon intercalation of the NBTI's LHS moiety. Among the considered NBTIs **A01-A18** (Figure S3c), most of them show stability (~0.0-1.5 Å), with the exception of **A13** and **A17** that display fluctuation probably because of the RHS rotation in the NBTI's binding site. The compactness of *apo* and NBTIs-ligated gyrase systems was assessed by calculating their radius of gyration (Rg), showing no significant deviations between all studied systems (Figure S3d).

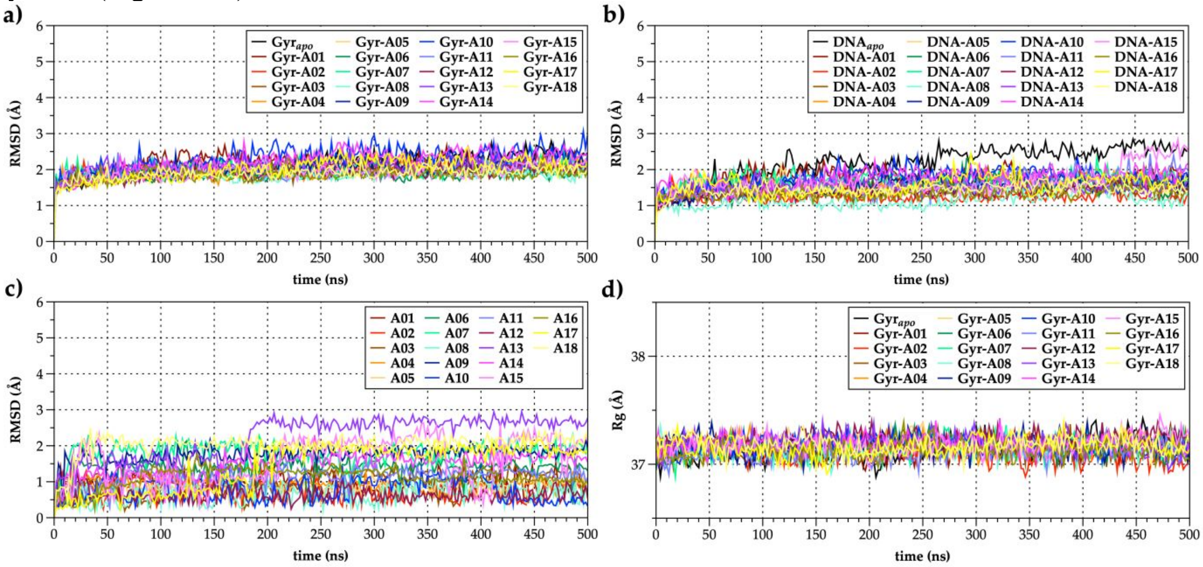

Figure S3. Plots of root-mean-square deviation (RMSD [Å]) and radius of gyration (Rg [Å]) of 500 ns MD simulations for *S. aureus* DNA gyrase *apo* form (ligand-free) and DNA gyrase complexes (PDB ID: 6Z1A) with 18 selected NBTI$_{SA}$ ligands (**A01-A18**). **a)** Protein backbone RMSD; **b)** DNA RMSD; **c)** Ligands RMSD; **d)** Rg plots.

Table S3. Statistical data of all six LIE methods employed (***cpptraj**$_{no\ sasa}$* & ***cpptraj**$_{sasa}$*, **NAMD**$_{no\ sasa}$ & **NAMD**$_{sasa}$, and **LIEW**$_{no\ sasa}$ & **LIEW**$_{sasa}$) calculated from the MD production trajectories sampled at four different time ranges (e.g., 100-200 ns, 250-350 ns, 400-500 ns, and 20-500 ns) for the training and the test set. The color scheme on the shaded areas corresponds to the color bars in Figure 10.

a) LIE method by not considering SASA parameter "*cpptraj*$_{no\ sasa}$"

TRAINING SET

	A01	A02	A03	A04	A05	A07	A08	A09	A13	A14	A15	A16	A17	A18
$\Delta G^{\circ}_{bind\_exp}$	**-8.31**	**-8.68**	**-10.34**	**-11.31**	**-11.03**	**-7.39**	**-11.23**	**-10.67**	**-7.79**	**-8.07**	**-8.64**	**-8.97**	**-9.57**	**-9.92**
**Time range (ns)**														
**100-200**	-9.77	-8.71	-9.08	-10.12	-9.36	-7.73	-11.30	-9.56	-8.31	-9.18	-8.72	-9.55	-9.73	-10.79
**250- 350**	-8.46	-8.90	-8.88	-9.69	-9.93	-8.08	-10.84	-9.37	-8.06	-9.58	-9.32	-10.32	-9.97	-10.50
**400- 500**	-8.57	-8.65	-8.83	-10.06	-10.40	-7.66	-10.23	-9.86	-8.01	-10.21	-9.09	-9.77	-10.27	-10.27
**20-500**	-8.67	-8.70	-9.05	-10.03	-10.07	-7.38	-11.17	-9.64	-8.10	-9.62	-8.82	-9.86	-10.06	-10.72
$\overline{X}$	**-8.87**	**-8.74**	**-8.96**	**-9.97**	**-9.94**	**-7.71**	**-10.89**	**-9.61**	**-8.12**	**-9.65**	**-8.99**	**-9.87**	**-10.01**	**-10.57**
$\sum(x_i - \overline{x})^2$	1.10	0.03	0.05	0.11	0.57	0.25	0.68	0.13	0.05	0.54	0.22	0.32	0.15	0.16
**SD**	**0.52**	**0.09**	**0.11**	**0.17**	**0.38**	**0.25**	**0.41**	**0.18**	**0.12**	**0.37**	**0.23**	**0.28**	**0.19**	**0.20**

TEST SET

	A06	A10	A11	A12
$\Delta G^{\circ}_{bind\_exp}$	**-10.89**	**-10.43**	**-10.26**	**-10.16**
**Time range (ns)**				
**100-200**	-10.40	-10.36	-10.34	-10.53
**250-350**	-9.92	-10.38	-10.16	-11.19
**400-500**	-10.66	-11.22	-10.71	-11.32
**20-500**	-10.72	-10.85	-10.31	-11.25
$\overline{X}$	**-10.43**	**-10.70**	**-10.38**	**-11.07**
$\sum(x_i - \overline{x})^2$	0.40	0.51	0.16	0.40
**SD**	**0.31**	**0.36**	**0.20**	**0.32**

$\overline{X}$: mean of the $\Delta G^{\circ}_{bind\_pred}$ values of four different time ranges from MD trajectories; $\Sigma(x_i - \overline{x})^2$: sum of square difference of each value and the mean; SD: standard deviation. All binding free energy values are in kcal/mol.

b) LIE method by considering SASA parameter "*cpptraj*$_{sasa}$"

		A01	A02	A03	A04	A05	A07	A08	A09	A13	A14	A15	A16	A17	A18
TRAINING SET	$\Delta G^{\circ}_{bind\_exp}$	**-8.31**	**-8.68**	**-10.34**	**-11.31**	**-11.03**	**-7.39**	**-11.23**	**-10.67**	**-7.79**	**-8.07**	**-8.64**	**-8.97**	**-9.57**	**-9.92**
	**Time range (ns)**														
	**100-200**	-9.39	-9.05	-9.71	-10.70	-10.32	-8.08	-11.37	-8.87	-7.56	-8.14	-8.59	-9.45	-10.25	-10.41
	**250-350**	-8.77	-9.27	-9.42	-10.59	-10.86	-8.50	-11.27	-9.09	-7.28	-8.03	-8.80	-9.65	-10.24	-10.14
	**400-500**	-8.66	-9.03	-9.35	-10.72	-11.38	-8.00	-10.66	-9.45	-7.50	-8.49	-8.45	-9.29	-10.74	-10.17
	**20-500**	-8.75	-9.00	-9.59	-10.67	-10.86	-7.90	-11.34	-9.18	-7.44	-8.37	-8.52	-9.49	-10.40	-10.39
	$\overline{X}$	**-8.89**	**-9.09**	**-9.52**	**-10.67**	**-10.86**	**-8.12**	**-11.16**	**-9.15**	**-7.45**	**-8.26**	**-8.59**	**-9.47**	**-10.41**	**-10.28**
	$\sum(x_i - \bar{x})^2$	0.34	0.04	0.08	0.01	0.56	0.21	0.34	0.17	0.04	0.13	0.07	0.06	0.17	0.06
	**SD**	**0.29**	**0.10**	**0.14**	**0.05**	**0.37**	**0.23**	**0.29**	**0.21**	**0.11**	**0.18**	**0.13**	**0.13**	**0.20**	**0.12**

		A06	A10	A11	A12
TEST SET	$\Delta G^{\circ}_{bind\_exp}$	**-10.89**	**-10.43**	**-10.26**	**-10.16**
	**Time range (ns)**				
	**100-200**	-10.05	-10.21	-10.73	-11.38
	**250-350**	-9.94	-10.30	-10.83	-12.43
	**400-500**	-10.50	-10.71	-11.34	-12.88
	**20-500**	-10.43	-10.45	-10.81	-12.19
	$\overline{X}$	**-10.23**	**-10.42**	**-10.93**	**-12.22**
	$\sum(x_i - \bar{x})^2$	0.23	0.14	0.23	1.19
	**SD**	**0.24**	**0.19**	**0.24**	**0.54**

$\overline{X}$: mean of the $\Delta G^{\circ}_{bind\_pred}$ values of four different time ranges from MD trajectories; $\Sigma(x_i - \bar{x})^2$: sum of square difference of each value and the mean; SD: standard deviation. All binding free energy values are in kcal/mol.

c) LIE method by not considering SASA parameter "NAMD$_{no\ sasa}$"

		A01	A02	A03	A04	A05	A07	A08	A09	A13	A14	A15	A16	A17	A18
TRAINING SET	$\Delta G^{\circ}_{bind\_exp}$	**-8.31**	**-8.68**	**-10.34**	**-11.31**	**-11.03**	**-7.39**	**-11.23**	**-10.67**	**-7.79**	**-8.07**	**-8.64**	**-8.97**	**-9.57**	**-9.92**
	**Time range (ns)**														
	**100-200**	-8.78	-9.48	-9.58	-9.95	-9.65	-7.20	-10.11	-9.03	-8.54	-9.50	-9.63	-9.66	-10.19	-10.62
	**250-350**	-8.96	-9.44	-9.56	-9.85	-9.38	-7.66	-9.86	-9.25	-8.37	-9.59	-10.26	-9.70	-9.55	-10.47
	**400-500**	-9.14	-9.35	-9.44	-9.46	-9.52	-7.93	-9.82	-9.09	-8.88	-9.69	-10.24	-9.56	-9.67	-10.09
	**20-500**	-8.82	-9.39	-9.52	-9.69	-9.52	-7.60	-10.05	-9.09	-8.51	-9.75	-10.06	-9.61	-9.84	-10.44
	$\bar{X}$	**-8.93**	**-9.41**	**-9.52**	**-9.74**	**-9.52**	**-7.60**	**-9.96**	**-9.12**	**-8.58**	**-9.63**	**-10.05**	**-9.63**	**-9.81**	**-10.41**
	$\sum(x_i - \bar{x})^2$	0.08	0.01	0.01	0.14	0.04	0.27	0.06	0.02	0.14	0.04	0.26	0.01	0.23	0.15
	**SD**	**0.14**	**0.05**	**0.05**	**0.18**	**0.09**	**0.26**	**0.12**	**0.08**	**0.19**	**0.10**	**0.26**	**0.05**	**0.24**	**0.19**

		A06	A10	A11	A12
TEST SET	$\Delta G^{\circ}_{bind\_exp}$	**-10.89**	**-10.43**	**-10.26**	**-10.16**
	**Time range (ns)**				
	**100-200**	-10.02	-9.99	-9.99	-9.97
	**250-350**	-10.38	-10.02	-10.10	-10.38
	**400-500**	-10.15	-10.26	-10.03	-10.14
	**20-500**	-10.30	-10.36	-10.10	-10.18
	$\bar{X}$	**-10.21**	**-10.16**	**-10.06**	**-10.17**
	$\sum(x_i - \bar{x})^2$	0.08	0.10	0.01	0.09
	**SD**	**0.14**	**0.16**	**0.05**	**0.15**

$\bar{X}$: mean of the $\Delta G^{\circ}_{bind\_pred}$ values of four different time ranges from MD trajectories; $\Sigma(x_i - \bar{x})^2$: sum of square difference of each value and the mean; SD: standard deviation. All binding free energy values are in kcal/mol.

d) LIE method by considering SASA parameter “NAMD$_{sasa}$”

TRAINING SET

	A01	A02	A03	A04	A05	A07	A08	A09	A13	A14	A15	A16	A17	A18
$\Delta G^{\circ}_{bind\_exp}$	**-8.31**	**-8.68**	**-10.34**	**-11.31**	**-11.03**	**-7.39**	**-11.23**	**-10.67**	**-7.79**	**-8.07**	**-8.64**	**-8.97**	**-9.57**	**-9.92**
**Time range (ns)**														
**100-200**	-8.75	-9.47	-9.26	-10.13	-9.65	-7.24	-9.95	-9.37	-8.43	-9.58	-9.69	-9.38	-10.24	-10.75
**250-350**	-9.00	-9.41	-9.50	-9.88	-9.40	-7.61	-9.73	-9.43	-8.40	-9.64	-10.25	-9.59	-9.51	-10.55
**400-500**	-8.93	-9.31	-9.32	-9.51	-9.71	-8.12	-10.46	-8.57	-8.96	-8.89	-9.99	-9.96	-9.87	-10.28
**20-500**	-8.81	-9.38	-9.47	-9.70	-9.53	-7.58	-9.98	-9.22	-8.51	-9.80	-10.11	-9.53	-9.82	-10.47
$\bar{X}$	**-8.87**	**-9.39**	**-9.39**	**-9.81**	**-9.57**	**-7.64**	**-10.03**	**-9.15**	**-8.58**	**-9.48**	**-10.01**	**-9.61**	**-9.86**	**-10.51**
$\sum(x_i - \bar{x})^2$	0.04	0.01	0.04	0.21	0.06	0.40	0.28	0.47	0.21	0.48	0.17	0.18	0.27	0.12
**SD**	**0.10**	**0.06**	**0.10**	**0.23**	**0.12**	**0.32**	**0.27**	**0.34**	**0.23**	**0.35**	**0.20**	**0.21**	**0.26**	**0.17**

TEST SET

	A06	A10	A11	A12
$\Delta G^{\circ}_{bind\_exp}$	**-10.89**	**-10.43**	**-10.26**	**-10.16**
**Time range (ns)**				
**100-200**	-10.03	-10.44	-10.33	-9.95
**250-350**	-10.35	-10.20	-10.19	-10.39
**400-500**	-10.19	-9.52	-9.67	-10.23
**20-500**	-10.29	-10.53	-10.18	-10.19
$\bar{X}$	**-10.22**	**-10.17**	**-10.09**	**-10.19**
$\sum(x_i - \bar{x})^2$	0.06	0.63	0.25	0.10
**SD**	**0.12**	**0.40**	**0.25**	**0.16**

$\bar{X}$: mean of the $\Delta G^{\circ}_{bind\_pred}$ values of four different time ranges from MD trajectories; $\Sigma(x_i - \bar{x})^2$: sum of square difference of each value and the mean; SD: standard deviation. All binding free energy values are in kcal/mol.

e) LIE method by not considering SASA parameter "LIEW $_{no\ sasa}$"

TRAINING SET

	A01	A02	A03	A04	A05	A07	A08	A09	A13	A14	A15	A16	A17	A18
$\Delta G^{\circ}_{bind\_exp}$	**-8.31**	**-8.68**	**-10.34**	**-11.31**	**-11.03**	**-7.39**	**-11.23**	**-10.67**	**-7.79**	**-8.07**	**-8.64**	**-8.97**	**-9.57**	**-9.92**
**Time range (ns)**														
**100-200**	-9.15	-9.38	-9.46	-9.60	-9.43	-8.55	-10.14	-8.52	-8.60	-9.58	-9.67	-9.56	-10.10	-10.17
**250-350**	-8.97	-9.29	-9.35	-9.59	-9.42	-8.38	-10.13	-8.85	-8.48	-9.76	-10.07	-9.59	-9.68	-10.34
**400-500**	-9.14	-9.34	-9.42	-9.49	-9.54	-8.45	-9.92	-8.85	-8.59	-9.94	-9.98	-9.57	-9.66	-10.00
**20-500**	-8.94	-9.26	-9.35	-9.52	-9.47	-8.42	-10.21	-8.73	-8.61	-9.73	-9.97	-9.53	-9.91	-10.26
$\bar{X}$	**-9.05**	**-9.32**	**-9.40**	**-9.55**	**-9.47**	**-8.45**	**-10.10**	**-8.73**	**-8.57**	**-9.75**	**-9.92**	**-9.57**	**-9.84**	**-10.19**
$\sum(x_i-\bar{x})^2$	0.04	0.01	0.01	0.01	0.01	0.02	0.05	0.07	0.01	0.07	0.09	0.00	0.13	0.07
**SD**	**0.10**	**0.05**	**0.05**	**0.05**	**0.05**	**0.06**	**0.11**	**0.13**	**0.05**	**0.13**	**0.15**	**0.02**	**0.18**	**0.13**

TEST SET

	A06	A10	A11	A12
$\Delta G^{\circ}_{bind\_exp}$	**-10.89**	**-10.43**	**-10.26**	**-10.16**
**Time range (ns)**				
**100-200**	-9.81	-10.32	-9.78	-9.81
**250-350**	-9.93	-10.45	-9.86	-10.13
**400-500**	-9.99	-10.46	-9.80	-9.97
**20-500**	-10.11	-10.77	-9.81	-10.07
$\bar{X}$	**-9.96**	**-10.50**	**-9.81**	**-10.00**
$\sum(x_i-\bar{x})^2$	0.05	0.11	0.00	0.06
**SD**	**0.11**	**0.17**	**0.03**	**0.12**

$\bar{X}$: mean of the $\Delta G^{\circ}_{bind\_pred}$ values of four different time ranges from MD trajectories; $\Sigma(x_i-\bar{x})^2$: sum of square difference of each value and the mean; SD: standard deviation. All binding free energy values are in kcal/mol.

f) LIE method by considering SASA parameter "LIEW$_{sasa}$"

TRAINING SET

	A01	A02	A03	A04	A05	A07	A08	A09	A13	A14	A15	A16	A17	A18
$\Delta G^{\circ}_{bind\_exp}$	**-8.31**	**-8.68**	**-10.34**	**-11.31**	**-11.03**	**-7.39**	**-11.23**	**-10.67**	**-7.79**	**-8.07**	**-8.64**	**-8.97**	**-9.57**	**-9.92**
**Time range (ns)**														
**100-200**	-9.15	-9.35	-9.51	-9.57	-9.44	-8.59	-10.21	-8.48	-8.60	-9.57	-9.64	-9.59	-10.08	-10.12
**250-350**	-9.00	-9.32	-9.38	-9.58	-9.42	-8.33	-10.04	-8.92	-8.46	-9.75	-10.12	-9.57	-9.63	-10.38
**400-500**	-8.88	-9.33	-9.20	-9.71	-9.62	-8.89	-10.54	-8.40	-8.55	-9.47	-9.68	-9.51	-10.01	-10.11
**20-500**	-8.94	-9.25	-9.36	-9.51	-9.48	-8.44	-10.24	-8.71	-8.61	-9.72	-9.95	-9.55	-9.91	-10.25
$\bar{X}$	**-8.99**	**-9.31**	**-9.36**	**-9.59**	**-9.49**	**-8.56**	**-10.26**	**-8.63**	**-8.56**	**-9.63**	**-9.85**	**-9.55**	**-9.91**	**-10.21**
$\sum(x_i - \bar{x})^2$	0.04	0.01	0.05	0.02	0.02	0.18	0.13	0.17	0.01	0.05	0.15	0.00	0.12	0.05
**SD**	**0.10**	**0.04**	**0.11**	**0.07**	**0.08**	**0.21**	**0.18**	**0.20**	**0.06**	**0.11**	**0.20**	**0.03**	**0.17**	**0.11**

TEST SET

	A06	A10	A11	A12
$\Delta G^{\circ}_{bind\_exp}$	**-10.89**	**-10.43**	**-10.26**	**-10.16**
**Time range (ns)**				
**100-200**	-9.81	-10.28	-9.77	-9.80
**250-350**	-9.83	-10.51	-9.95	-10.14
**400-500**	-9.95	-9.94	-9.34	-10.10
**20-500**	-10.15	-10.79	-9.84	-10.12
$\bar{X}$	**-9.94**	**-10.38**	**-9.72**	**-10.04**
$\sum(x_i - \bar{x})^2$	0.07	0.39	0.22	0.08
**SD**	**0.13**	**0.31**	**0.23**	**0.14**

$\bar{X}$: mean of the $\Delta G^{\circ}_{bind\_pred}$ values of four different time ranges from MD trajectories; $\Sigma(x_i - \bar{x})^2$: sum of square difference of each value and the mean; SD: standard deviation. All binding free energy values are in kcal/mol.

Table S4. Correlation of experimental ($\Delta G^{\circ}_{bind\_exp}$) and predicted ($\Delta G^{\circ}_{bind\_pred}$) binding free energy values predicted by the selected VMD's NAMD Energy plugin without considering SASA (**NAMD**$_{no\ sasa}$) calculated from the resulting MD production trajectories sampled at time range 20-500 ns. All binding free energy values are in kcal/mol.

**Training set**			**Test set**		
**ID**	$\Delta G^{\circ}_{bind\_exp}$	$\Delta G^{\circ}_{bind\_pred}$	**ID**	$\Delta G^{\circ}_{bind\_exp}$	$\Delta G^{\circ}_{bind\_pred}$
**A01**	-8.31	-8.82	**A06**	-10.89	-10.30
**A02**	-8.68	-9.39	**A10**	-10.43	-10.36
**A03**	-10.34	-9.52	**A11**	-10.26	-10.10
**A04**	-11.31	-9.69	**A12**	-10.16	-10.18
**A05**	-11.03	-9.52			
**A07**	-7.39	-7.60			
**A08**	-11.23	-10.05			
**A09**	-10.67	-9.09			
**A13**	-7.79	-8.51			
**A14**	-8.07	-9.75			
**A15**	-8.64	-10.06			
**A16**	-8.97	-9.61			
**A17**	-9.57	-9.84			
**A18**	-9.92	-10.44			

Molecular re-docking validation data

Table S5. Molecular re-docking validation data.

Complex	RMDS (Å)		
	Docked pose 1	Docked pose 2	Docked pose 3
S. aureus DNA gyrase-DNA-AMK12 (PDB ID: 6Z1A) [2]	0.8324	1.7501	1.3039
E. coli DNA gyrase-DNA-gepotidacin (PDB ID: 6RKS) [3]	0.9880	1.6198	1.3691

RMSD (heavy-atoms root-mean-square deviation) values in Angstrom (Å) units calculated between each calculated dock pose and its natively present co-crystallized (AMK12), i.e., cryo-EM (gepotidacin) ligand.

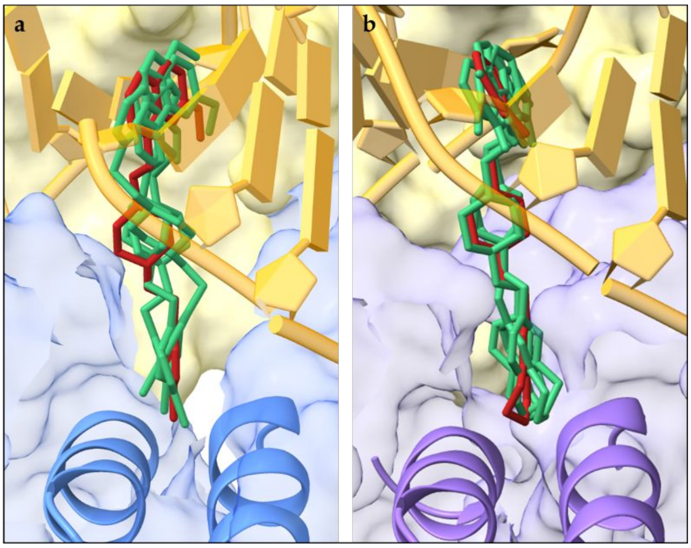

Figure S4. Ligands reproduction obtained by re-docking of the natively present ligands conformation (AMK12 and gepotidacin, respectively) and their calculated docked poses in a) *S. aureus* DNA gyrase-AMK12 crystal structure complex (PDB ID: 6Z1A [2]); GyrA subunit os colored in blue, while GyrB in yellow, and in b) *E. coli* DNA gyrase-gepotidacin cryo-EM complex (PDB ID: 6RKS [3]). *S. aureus* GyrA and *E. coli* GyrA subunit (cartoon representation) are colored in blue and violet, respectively, while GyrB subunit in both enzymes (surface representation) in yellow. The experimental co-crystallized AMK12 and gepotidacin conformations are represented in dark red, while their re-docked poses are in light green (stick representation).

References

(1) Shrake, A.; Rupley, J. A. Environment and Exposure to Solvent of Protein Atoms. Lysozyme and Insulin. *J. Mol. Biol.* **1973**, *79* (2), 351–371. https://doi.org/10.1016/0022-2836(73)90011-9.

(2) Kolarič, A.; Germe, T.; Hrast, M.; Stevenson, C. E. M.; Lawson, D. M.; Burton, N. P.; Vörös, J.; Maxwell, A.; Minovski, N.; Anderluh, M. Potent DNA Gyrase Inhibitors Bind Asymmetrically to Their Target Using Symmetrical Bifurcated Halogen Bonds. *Nat. Commun.* **2021**, *12* (1), 1–13.

(3) Vanden Broeck, A.; Lotz, C.; Ortiz, J.; Lamour, V. Cryo-EM Structure of the Complete E. Coli DNA Gyrase Nucleoprotein Complex. *Nat. Commun.* **2019**, *10* (1), 1–12.
